# Dielectric Measurement of Agricultural Grain Moisture—Theory and Applications

**DOI:** 10.3390/s22062083

**Published:** 2022-03-08

**Authors:** Scott B. Jones, Wenyi Sheng, Dani Or

**Affiliations:** 1 Department of Plants, Soils, and Climate, Utah State University, Logan, UT 84322, USA; scott.jones@usu.edu; 2 Key Laboratory of Smart Agriculture Systems, China Agricultural University, Ministry of Education, Beijing 100083, China; 3 Division of Hydrologic Sciences, Desert Research Institute, Reno, NV 89512, USA; dani.or@dri.edu

**Keywords:** cereal grain, moisture content, complex permittivity, dielectric mixture model, bound water

## Abstract

Moisture content is a critical variable for the harvesting, processing, storing and marketing of cereal grains, oilseeds and legumes. Efficient and accurate determination of grain moisture content even with advanced nondestructive techniques, remains a challenge due to complex water-retaining biological structures and hierarchical composition and geometry of grains that affect measurement interpretation and require specific grain-dependent calibration. We review (1) the primary factors affecting permittivity measurements used in practice for inferring moisture content in grains; (2) develop novel methods for estimating critical parameters for permittivity modeling including packing density, porosity, water binding surface area and water phase permittivity and (3) represent the permittivity of packs of grains using dielectric mixture theory as a function of moisture content applied to high moisture corn (as a model grain). Grain permittivity measurements are affected by their free and bound water contents, chemical composition, temperature, constituent shape, phase configuration and measurement frequency. A large fraction of grain water is bound exhibiting reduced permittivity compared to that of free water. The reduced mixture permittivity and attributed to hydrophilic surfaces in starches, proteins and other high surface area grain constituents. The hierarchal grain structure (i.e., kernel, starch grain, lamella, molecule) and the different constituents influence permittivity measurements due to their layering, geometry (i.e., kernel or starch grain), configuration and water-binding surface area. Dielectric mixture theory offers a physically-based approach for modeling permittivity of agricultural grains and similar granular media.

## 1. Introduction

### 1.1. Moisture Determination in Agricultural Grains

Determination of the moisture content of cereal grains, oilseeds and legumes is crucial in three respects. (1) From a technological standpoint, harvesting, drying, storing and milling operations are all based on grain moisture levels. (2) Analytically, all methods for moisture content determination are related to dry matter or a standard moisture content. (3) In commercial applications, the pricing of grain is a function of moisture content throughout the entire marketing process. Moisture contents at harvest may be about twice the desirable storage moisture value, with a few exceptions such as high moisture content crops harvested for seed or oil. Forced drying of grains is frequently needed to reduce harvest moisture levels to near storage values, this commonly used practice requires large energy inputs. These additional energy costs and attainment of safe storage moisture state can benefit from the ability to obtain more accurate and reliable moisture determination. The financial importance of improved moisture content determination is obvious whenever excess moisture damages grain via fungi, mold, etc. [1] and can be further illustrated by considering a variation of 1 percentage point in estimating the moisture content of a 100,000-ton shipment resulting in a 1000-ton difference valued at around $100,000 [2].

Measurement of the permittivity of porous and granular media is rapidly advancing, with novel, well-calibrated and characterized meters and devices [3] offer fast and efficient measurement of moisture content at reasonable costs [4,5]. In discussing the modeling and measurements, we will refer to permittivity as the relative complex permittivity and to its real and imaginary components as the dielectric constant and loss factor, respectively. The effectiveness of the permittivity measurement method relies on the large disparity between the permittivity of water in a “free” state, which has a relatively large permittivity of *ε*_w_ ≈ 80, and that of many solid constituents [6], which generally have a permittivity of from 3 to 5. A primary difficulty with these measurement methods is that a portion of the water within granular and porous materials that exhibit large surface areas (i.e., starch and proteins) is tightly bound to the solid surfaces [7,8,9]. Additionally, the water retained in these materials may be confined within the solid and air phases in a variety of geometrical configurations [10,11]. The first few bound water molecule layers are rotationally hindered and hence unable to respond to the imposed alternating electric field. Consequently, the bound portion of water relaxes at a lower frequency than free water and exhibits a permittivity value closer to that of solid constituents. The consequence is a reduction in the effective permittivity of the porous medium leading to erroneous water content estimation. Biological materials such as cereal grains are composed of a complex structure and bind relatively large amounts of water, which is especially true for moisture levels after harvest.

In the following we focus on high moisture corn, where determination of its water content has historically been error-prone relative to grain harvested at lower moisture contents [12,13,14]. Hurburgh et al. [15] evaluated corn moisture measurement accuracy and concluded that studies of grain composition with respect to dielectric properties offers the greatest potential for improving moisture meter accuracy. Hurburgh et al. [16] presented standard deviations of meter measurements relative to oven drying from 5-years of data for corn moisture contents of 10, 15, 20, 25 and 30 percent having moisture percentage standard deviations of 1.10, 0.59, 0.55, 1.05 and 1.64, respectively. Low accuracy in measuring cereal grain moisture content prompted the United States Congress to include in the 1990 Farm Bill a directive to the National Institute of Standards and Technology (NIST) to develop a program to reduce error and variability in moisture content measurements in the United States. Improved variety-dependent moisture meter calibrations, testing methods and technologies were suggested for more accurate determination of cereal grain moisture [17]. The first official dielectric type moisture meter (Motomco Model 919; Paterson, NJ, USA) was approved by the US government four decades ago in 1962. In 1998 almost 2 million official grain moisture inspections were carried out in the US alone, by federal, state and private inspection agencies on over 214 million metric tons of grains and oilseeds. The vast quantity of grain and oilseeds marketed worldwide accentuates the critical need for the advancement of the science and technologies aimed at improving the accuracy of moisture content determination in cereal grains and biological materials in general. This is especially true in developing countries where grain moisture determination often requires low-tech or alternative methods [18]. The minimum tolerance for U.S.D.A. Grain Inspection Packers and Stockyards Administration (GIPSA) approved grain moisture meters is ±0.8 percent moisture content for corn, oats, rice, sorghum and sunflower and ±0.7 percent for all other grains [19]. In 1999 a new official water content meter was adopted (Dickey-John GAC 2100), which provided improvements in speed and accuracy. However, commercial moisture meters continued to rely on the same moisture measurement algorithms used in past decades and in some cases used the same circuitry due to the costs involved in establishing new calibration equations needed for newer high-tech equipment [20]. A new unified grain moisture measurement algorithm was developed and introduced as a substantial improvement for grain moisture determination, focused on a measurement frequency of 149 MHz [20,21]. By 2009, there was momentum building to further improve the moisture measurement sensor technology to avoid errors such as those cited in the rough rice evaluations of meter vs. air oven reference measurements [22]. The 2021 National Conference on Weights and Measures Publication 14 details the technical policy, checklists and test procedures for grain moisture determination using meters and near infrared grain analyzers [3].

Much of the reported measurement accuracy of grain moisture meters has been carried out under controlled conditions in scientific laboratories, a question remains as to the measurement capability under ‘real world’ conditions where grain variety may deviate from calibration standards or where conditions are not well controlled. Since both temperature and packing density are routinely determined by moisture meters, other factors affecting permittivity of cereal grains such as constituent composition and form of water (i.e., bound or free) remains a topic of ongoing research [8,23,24,25,26]. Dielectric mixture theory offers a physically-based approach to account for the majority of factors influencing bulk grain permittivity measurements including water status, grain density, temperature, grain geometry, measurement frequency and configuration of the grain constituents. In the following we discuss these factors followed by a sampling of dielectric mixture models.

### 1.2. Factors Influencing Permittivity Measurements of Agricultural Grains

Since the 1950s, dielectric properties of cereal grains have been investigated [27] and used to infer their moisture content [28]. Seven decades later, a variety of measurement techniques are available [29] spanning a wide range of frequencies, including DC resistivity cell, parallel plate (<30 MHz; [30]) and coaxial cell (200 MHz–20 GHz; [31]), transmission line (500 MHz to 110 GHz; [32]), resonant cavity (500 MHz to 110 GHz; [33]) and free space measurement techniques [34,35]. Virtually all of these techniques have been applied to dielectric measurement of cereal grains (See review by Nelson [36]). Permittivity measurement methods are genotype dependent, requiring grain-dependent calibration and generally rely on empirical expressions for moisture content interpretation for each type of grain measured.

The use of permittivity measurement for moisture determination in cereal grains and other porous materials is subject to biological, chemical, physical and environmental factors [13,37,38], which hamper accuracy. Some of the factors that need to be considered in such measurements include the dependence of the permittivity, ε, on water content [38], electromagnetic measurement frequency [39], energy of water (i.e., bound or free) [40], bulk density or porosity [41,42,43], temperature [8,44,45,46], chemical composition [47], particle shape [11,29] and pH [48].

#### 1.2.1. Water Content and the Grain Aqueous Phase

Water content is the major factor of interest and has the most significant effect on the measured permittivity of cereal grains, which have been measured over frequency ranges from 250 Hz to 12 GHz [13]. Moisture content varies both spatially and temporally on the ear or head [49] and within the kernel [50]. The water content is made up of that which is bound to solid surfaces, and at high enough water contents a portion may exist in a relatively free state within the grain. Distinguishing between bound and free water is not trivial, nor is the division between the two well defined. Decades of research attempting to define and characterize bound water have produced a plethora of phrases such as tightly- or loosely-bound [51], monolayer or multilayer [52,53], vicinal [54], adsorbed [55,56], solid-like [57], irrotational or rotationally hindered [58,59], hydration [60,61], critical hydration [62] and other such terms. This is further complicated by a long list of methods used to determine bound water content, some of which are listed by [63], including calorimetry, dilatometry, freeze drying, freezing point depression, vapor point depression, dielectric absorption, infrared (IR), nuclear magnetic resonance (NMR), electron spin resonance (ESR), ultraviolet, phosphorescence and sorption techniques. Other methods include thermally stimulated depolarization currents (TSDC), differential scanning calorimetry (DSC) [64] and dielectric relaxation spectroscopy (DSR) [65]. Despite the various measurement methods, it seems that the entire amount of water within the grain from harvest to storage moisture levels (e.g., 0.20 to 0.11 dry-basis (db)) is in a bound state (see discussion in Section 2.3). An exception occurs for corn harvested at higher than usual moisture levels (i.e., up to *M*_db_ = 0.3) where a combination of bound and free water results in more complex water content-permittivity relations. Since water content determination is the goal of our permittivity measurements, much of this review is devoted to defining or estimating the bound-free water relations.

The generally preferred format for reporting moisture content of grains and legumes is on a wet-basis, *M*_wb_, while constituent water content is often reported on a dry-basis, *M*_db_. For dielectric mixture theory and most electromagnetic-based sensing methods, we are interested in volumetric constituent quantities and require the volumetric moisture content, *M*_v_ (e.g., volume of water per bulk grain volume), which we obtain from either the dry or wet-basis moisture content using the grain bulk density, *ρ*_b_, and the density of free water, *ρ*_w_, as follows

(1) $$\begin{array}{c} M_{v}=M_{\mathrm{db}}\frac{\rho_{b}}{\rho_{w}}=\left(\frac{M_{\mathrm{wb}}}{1-M_{\mathrm{wb}}}\right)\frac{\rho_{b}}{\rho_{w}} \end{array}$$

#### 1.2.2. Dielectric Measurement Frequency

**Figure 1:**
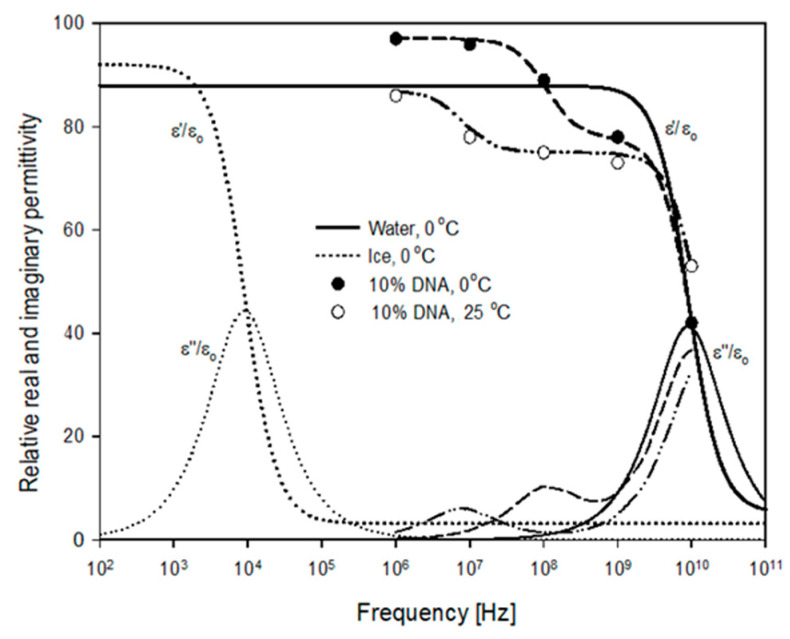
Dielectric constant (*ε*′) and loss factor (*ε*″) spectra of water and ice at 0 °C and with digitized and modeled spectra of DNA solution at 0 and at 25 °C [66].

Measurement frequency influences permittivity determination and contains insights into the relaxation phenomenon associated with constituent size and shape [12,13]. Under the influence of an alternating electric field, molecules tend to rotate and become aligned with the applied field. Consequently, electric energy may be either stored (measured as permittivity) by molecules and electrons which rotate and are perturbed from their equilibrium positions, or electric energy may be dissipated as a conductive loss due to the migration of ions (charges). The oscillatory nature of an alternating current creates a cycling of the field direction, which becomes faster with increasing frequency. At a certain frequency, molecules can no longer follow the electric field resulting in a reduction of the energy storage potential (permittivity) in a process known as dielectric relaxation. This phenomenon is illustrated in Figure 1 for ice and free water at 0 °C, where both dielectric constant and loss factor are plotted. The relaxation frequency is a function of the molecule sizes, mobility, polarizability and other factors.

Kuntz [67] suggested that when measuring the frequency-dependent permittivity of a protein solution (ignoring Hz to kHz frequencies where other low-frequency complications exist) one encounters three regions of rapidly changing permittivity with frequency at ranges of 10^5^–10^7^ Hz, 10^8^–10^9^ Hz and near 2 × 10^10^ Hz [68]. The lowest frequency region is a function of the molecular weight and the dielectric relaxation or dispersion is a result of the tumbling motion of the macromolecule. The midrange frequency is bound water related, being due to the partially-hindered rotation of water molecules tightly bound to the protein molecule. The highest region represents the dispersion of the free water molecules in the system.

Dilute mixtures of DNA and Protein in water exhibit both bound- and free-water relaxations as pictured in Figure 1. Fukuzaki et al. [66] demonstrated the effect of increasing polymer (albumin) concentrations (5 to 20 percent) which resulted in increased relaxation frequency (850–290 kHz) due to the molecule while relaxation frequencies of bound (63–81 MHz) and free water (17.8–18.3 GHz) were relatively constant. Mashimo and Miura [69] showed a unit inverse relation (log-log scale) of the relaxation time of the lowest frequency process (between 10 and 30 MHz) with the molecular weight of 10 different proteins. Relaxations resulting from the combined constituents of grains and other food substances exhibit a wide spread in relaxation frequencies extending from molecular relaxations (kHz) through bound- and free-water relaxations (MHz to GHz) [47].

Measurement frequency is also a factor when considering the relative size of inclusions or layering for modeling dielectric behavior of a mixture of constituents. The analysis of Chan and Knight [70] is based on the ratio of the wavelength (*λ*) to layer (heterogeneity) thickness (*t*), *λ*/*t*, and provides insight into this type of phenomenon. For *λ*/*t* > 10 the dielectric constant of the soil is computed as an arithmetic average (effective medium theory) of the layers, while for *λ*/*t* < 1 the geometric average (Ray theory) of the soil layers is used to compute the soil dielectric constant. Scattering effects that occur within the transition zone, 1 < *λ*/*t* < 10, may cause measurement problems. With each of these scenarios the propagation direction of the EM wave relative to the layering is a significant factor. For cereal grains and legumes, the assumption of *λ*/*t* > 10 applies for frequencies less than 1 GHz and grain size less than 1 cm.

#### 1.2.3. Temperature Effects

The temperature dependence of the permittivity of grains can be attributed largely to the effect of temperature on the water phase. The temperature-dependent permittivity of free water may be described by the following empirical expression [71]:

(2) $$\varepsilon_{\mathrm{fw}}\left(T\right)=78.54\left[1-4.579\times 10^{-3}\left(T-298\right)+1.19\times 10^{-5}{\left(T-298\right)}^{2}-2.8\times 10^{-8}{\left(T-298\right)}^{3}\right]\;$$

where *T* (K) is the temperature in Kelvin. The temperature dependence on the bound portion of water is more complicated, especially since the distinction between bound and free water is vague. Or and Wraith [59] derived a temperature-dependent expression describing the bound water content (*M*_bw_) expressed in terms of the computed bound water layer thickness, *x*(*T*) (m), the specific surface area, *A*_s_ (m^2^ g^−1^), and the bulk density, *ρ*_b_ (g m^−3^), of the porous medium, written as:

(3) $$M_{\mathrm{bw}}=x\left(T\right)A_{s}\rho_{b}\;$$

where *x*(*T*) was derived from the viscosity profile of water as a function of distance from a clay surface coupled with the Debye Model [72] predicting relaxation frequency of a polar liquid, f* (Hz), (i.e., cutoff frequency of Or and Wraith [59]), below which bound water relaxes. In addition to the cutoff frequency, the assumed radius of the bound water molecule, *r* (m), has a significant effect on the resulting temperature-dependent bound water layer thickness, *x*(*T*) (m), computed as:

(4) $$x\left(T\right)=\frac{\alpha }{-d+T\cdot \ln(\frac{kT}{8\pi^{2}r^{3}cf^{*}})}\;$$

where the constants *α* = 1621 (Å K), *d* = 2.047 × 10^3^ (K), *c* = 9.5 × 10^−7^ (Pa s) and *κ* is the Boltzmann constant (1.38062 × 10^−23^ (J K^−1^)). The effect of temperature on the permittivity of water within porous materials (soils) was suggested by Or and Wraith [59] to be the result of an interplay between (1) the reduction in the permittivity of bulk or free water with increasing temperature, and (2) the increased permittivity brought about by the liberation of bound water to a less hindered state (i.e., greater permittivity). Similar trends were found in corn starch where a temperature increase produced an increase in the measured permittivity at low moisture contents, while permittivity decreased at higher moisture contents with increased temperature [73]. Tait et al. [57] showed an increase in the ratio of loosely bound water to that of solid-like (monolayer) water with increasing temperature from −40 to +20 °C for starch samples at 20 percent moisture content, providing further evidence for bound water liberation with increased temperature.

Bound water in soils exhibits behaviors similar to that in grains and other porous media. Activation energies for water on protein and carbohydrate surfaces are comparable to those bound to soil mineral surfaces (e.g., Fe_2_O_3_, clay). Pennock and Schwan [74] found an activation energy for water on hemoglobin of 7.3 kcal mol^−1^ (0.5 to 1 GHz). Fripiat et al. [75] suggested the activation energy for water on aerogel and glass powder to be 10 kcal mol^−1^. Roudaut et al. [76] found an activation energy in bread and starch of 12 kcal mol^−1^. Jia et al. [77] determined the activation energy for conversion of chemically bound water to lie in the range of 0.26 and 5.78 kcal mol^−1^. Mirzaee et al. [78] reported drying activation energies dependent on applied air velocities of between 7.01 to 8.07 kcal mol^−1^ in drying apricots. McCafferty et al. [79] found an activation energy of 16 kcal mol^−1^ for 2.5 layers of water on α-Fe_2_O_3_. These numbers suggest an average activation energy of roughly 5 to 10 kcal mol^−1^ for water bound in these porous media where variations in measured values may be attributable to differences in measurement techniques or to differences in water binding surface.

#### 1.2.4. The Role of Grain- and Constituent-Shape

**Figure 2:**
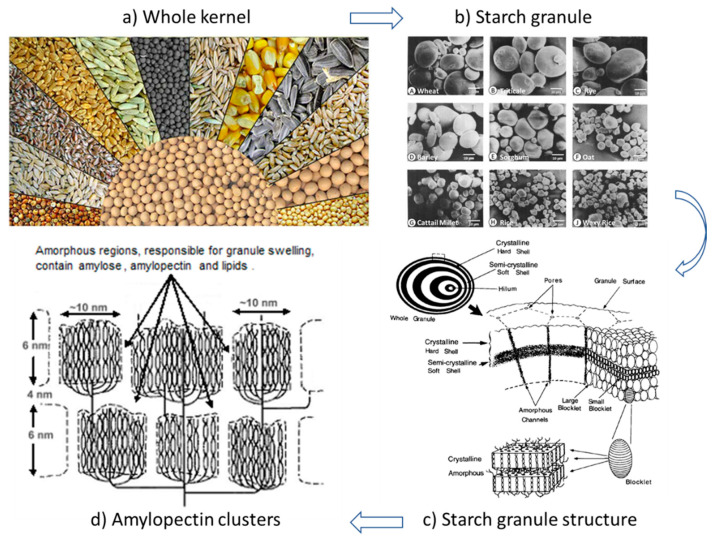
Hierarchy of shape and structure from (**a**) the kernel [87] to (**b**), the starch granule [84] to (**c**), layering within a granule and the alternate layering substructure [85] to (**d**), the highly branched and long chain polymers [85]. Images and permissions obtained from cited references.

The shape of any portion of grain from the kernel itself, to the starch granule, to the molecular components (Figure 2), may influence the dielectric measurement based on each component’s respective dipole moment. Particle shape effects have been measured on individual kernels [80] and on mixtures of isotropic [81,82] and anisotropic particles [11]. A spheroid (rotation ellipsoid) provides a convenient mathematical approximation for many of the shapes shown in Figure 2. We refer to the spheroid with respect to the rotation axis, a, and the common axes, b and c. Expressions which account for permittivity enhancement arising from the dipole interaction due to inclusion shape have been developed using the so-called depolarization factor, *N* [83]. Graphical and numerical approximations for *N* are available for individual geometries of disks, spheres and needles [83,84,85]. A continuous empirical function describing the depolarization factor ranging from a disk- to a needle-shaped spheroid was fitted by Jones and Friedman [11] as:

(5) $$N^{p}=\frac{1}{1+1.6\left(\frac{a}{b}\right)+0.4{\left(\frac{a}{b}\right)}^{2}}$$

(6) $$N^{n}=0.5\left(1-N^{a}\right)$$

where the depolarization factor for the electric field aligned parallel to the axis of symmetry (rotation axis) is *N*^p^ and is *N*^n^ when normal to the axis of symmetry and where *a*/*b* is the particle or kernel aspect ratio. Particle shape effects are limited to inclusions smaller than approximately one-sixth of the free-space wavelength [86], and therefore inclusion or grain size is a factor affecting measurement frequency and the resulting permittivity.

### 1.3. Dielectric Mixture Theory

Dielectric mixture theory approximates the effective permittivity of a mixture as a function of the constituent permittivities, their fractional volumes and other “microscopic” parameters (e.g., temperature, frequency, inclusion shape). Two-phase dielectric mixture models for predicting the permittivity of different grains and vegetables have been considered [47,88]. For the case of a two-phase system, discrete inclusions (kernels) of permittivity, *ε*_1_, are contained in a background host medium (air) of permittivity, *ε*_0_. A Clausius-Mossotti, Lorentz-Lorenz relation [89] is one example of a two-phase mixture model [90,91], given as:

(7) $$\frac{\varepsilon_{\mathrm{eff}}-\varepsilon_{0}}{\varepsilon_{\mathrm{eff}}+2\varepsilon_{0}}=\phi_{1}\frac{\varepsilon_{1}-\varepsilon_{0}}{\varepsilon_{1}+2\varepsilon_{0}}$$

where *ε*_eff_ is the effective permittivity of the mixture, which in practice is the measured value, and *φ*_1_ is the volume fraction of the inclusions or scatterers. When this formula is explicitly written in terms of the *ε*_eff_ it is known as the Maxwell-Garnett equation and is limited to dilute mixtures (i.e., *φ*_1_ << 1). Alternative implicit relations have been derived which better approximate mixture permittivities of large inclusion volume fraction (low porosity) by representing the background permittivity to be more like that of the background-inclusion mixture than that of the background alone. A more generalized mixture formula for an isotropic mixture of ellipsoidal inclusions has been used to describe a family of mixture models [92] given by:

(8) $$\varepsilon_{\mathrm{eff}}=\varepsilon_{0}+\frac{\sum_{i=p,n,n}\frac{\phi_{1}\left(\varepsilon_{1}-\varepsilon_{0}\right)\left[\varepsilon_{0}+v\left(\varepsilon_{\mathrm{eff}}-\varepsilon_{0}\right)\right]}{3\left[\varepsilon_{0}+v\left(\varepsilon_{\mathrm{eff}}-\varepsilon_{0}\right)+N^{i}\left(\varepsilon_{1}-\varepsilon_{0}\right)\right]}}{1-\sum_{i=p,n,n}\frac{\phi_{1}N^{i}\left(\varepsilon_{1}-\varepsilon_{0}\right)}{3\left[\varepsilon_{0}+v\left(\varepsilon_{\mathrm{eff}}-\varepsilon_{0}\right)+N^{i}\left(\varepsilon_{1}-\varepsilon_{0}\right)\right]}}\;$$

where the summation is taken over the three axial directions (i.e., parallel or normal to the ellipsoid rotation axis) and *ν* is a parameter giving the Maxwell-Garnett [91] mixture rule when equal to 0, the Polder and van Santen [93] mixture formula when *ν* = 1 − *N*^i^, and the “coherent potential” [94] mixture rule when *ν* = 1. The resulting effective permittivity of the mixture is strongly dependent on the particle shape (aspect ratio) and alignment (i.e., parallel or normal) with respect to the applied electric field.

The effective permittivity of an isotropic three-phase confocal system of ellipsoids was derived by Sihvola and Lindell (their Equation (48) after [95]) for any number of confocal ellipsoids, written as:

(9) $$\varepsilon_{\mathrm{eff}}=\varepsilon_{0}+\frac{\frac{\varepsilon_{0}}{3}\sum_{i=p,n,n}(\frac{n_{v}\alpha^{i}}{\varepsilon_{0}})}{1-\frac{1}{3}\sum_{i=p,n,n}N_{1}^{i}(\frac{n_{v}\alpha^{i}}{\varepsilon_{0}})}\;$$

where the polarizability term in parenthesis is given as a series expansion written here for a dual, confocal ellipsoid system as:

(10) $$\frac{n_{v}\alpha^{i}}{\varepsilon_{0}}\;=\;(\phi_{1}+\phi_{2})\frac{\left\{\left(\varepsilon_{1}-\varepsilon_{0}\right)+[\varepsilon_{1}+N_{1}^{i}\left(\varepsilon_{0}-\varepsilon_{1}\right)]\frac{\left(\varepsilon_{2}-\varepsilon_{1}\right)\frac{\phi_{2}}{\left(\phi_{1}+\phi_{2}\right)}}{\left[\varepsilon_{1}+N_{2}^{i}\left(\varepsilon_{2}-\varepsilon_{1}\right)\right]}\right\}}{\left\{[\varepsilon_{0}+N_{1}^{i}\left(\varepsilon_{1}-\varepsilon_{0}\right)]+N_{1}^{i}(1-N_{1}^{i})\left(\varepsilon_{1}-\varepsilon_{0}\right)\frac{\left(\varepsilon_{2}-\varepsilon_{1}\right)\frac{\phi_{2}}{\left(\phi_{1}+\phi_{2}\right)}}{\left[\varepsilon_{1}+N_{2}^{i}\left(\varepsilon_{2}-\varepsilon_{1}\right)\right]}\right\}}$$

where *ϕ*_1_ and *ϕ*_2_ are the volume fractions and *N*_1_*^i^* and *N*_2_*^i^* are the depolarization factors of the outer and inner ellipsoids, respectively. Six different combinations of the solid, liquid and gas phases are possible for a three-phase system.

The four-phase mixture formula of Dobson et al. [96] is based on deLoor’s theory [97] where solids act as the host phase with disk-like inclusions (*N*^p^ = 1, *N*^n^ = 0) of bound water, free water and air randomly distributed throughout the mixture written as:

(11) $$\varepsilon_{\mathrm{eff}}=\frac{3\varepsilon_{s}+2\phi_{\mathrm{fw}}\left(\varepsilon_{\mathrm{fw}}-\varepsilon_{s}\right)+2\phi_{\mathrm{bw}}\left(\varepsilon_{\mathrm{bw}}-\varepsilon_{s}\right)+2\phi_{a}\left(\varepsilon_{a}-\varepsilon_{s}\right)}{3+\phi_{\mathrm{fw}}\left(\frac{\varepsilon_{s}}{\varepsilon_{\mathrm{fw}}}-1\right)+\phi_{\mathrm{bw}}\left(\frac{\varepsilon_{s}}{\varepsilon_{\mathrm{bw}}}-1\right)+\phi_{a}\left(\frac{\varepsilon_{s}}{\varepsilon_{a}}-1\right)}\;$$

This mixture theory in combination with Equation (3) was used by [59] to model the interplay between free- and bound-water of soil. Separation of the water phases into bound and free water, can be advantageous for certain applications but measurement of surface area or other methods for estimating bound water must be used.

Isolating and characterizing the dependence of factors such as water content and status, measurement frequency, temperature and kernel shape on the permittivity of grains, seed and legumes is difficult and the biological nature adds additional complexity. Incorporating these effects into dielectric mixture models should improve the characterization of permittivity for water content determination. We have examined the makeup and physical characteristics of the principal components of grains and legumes to further the understanding of grain constituent influence on permittivity measurements.

To summarize, there is a need for more reliable, economical and accurate moisture measurement devices and dielectric measurements have increasingly greater potential as the technology advances. Increasing our understanding of the physical factors influencing and confounding such measurements should lead to improved and more accurate measurement capability. These factors include water content and status, measurement frequency, temperature, constituent shape, and packing density (covered in Section 2). Dielectric mixture theory provides a basis for modeling and estimating these effects and a number of models have been derived under different conditions for 2, 3 and 4 phase systems. Defining the physical properties of constituents is a necessary aspect in applying dielectric mixture theory.

## 2. Physical Properties of Agricultural Grains

In this section, we review the influence of constituents of some cereal grains and legumes on permittivity measurements by their volumetric presence and permittivities. We also consider the shape and packing of grains and legumes and the importance of the volume fraction of solids within the realm of the measurement, which is another critical and yet unresolved issue. We conclude this section by discussing aspects of water stored within the grain as well as constituent surface area influence on water status.

### 2.1. Grain Composition

**Figure 3:**
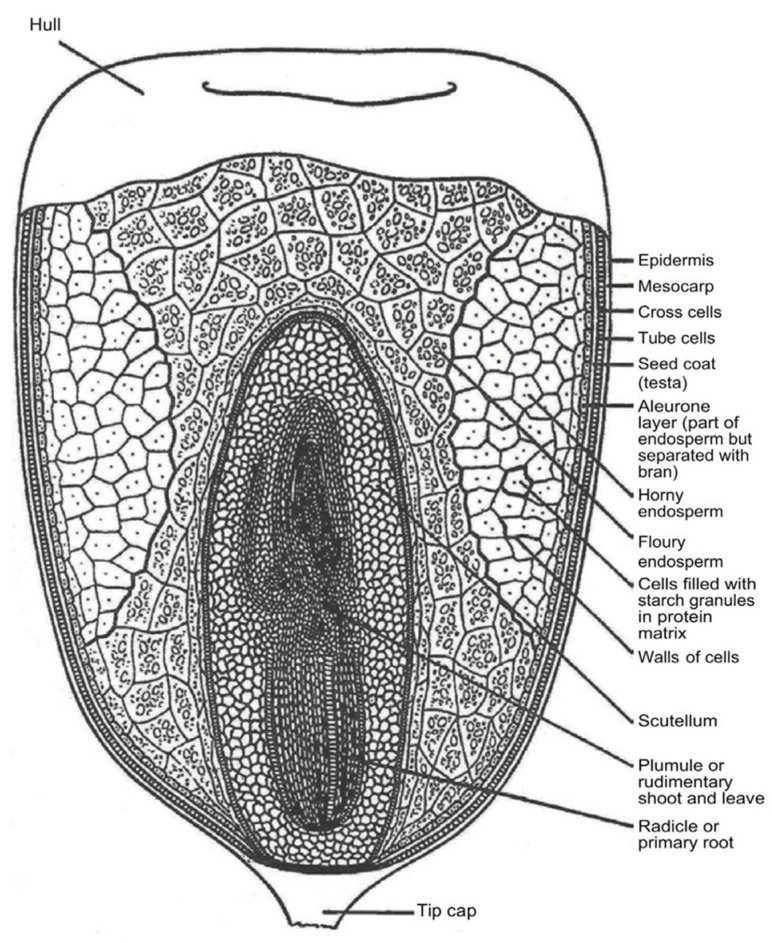
Longitudinal cross-section of corn kernel [98].

The complex composition of cereal grains is illustrated by the corn kernel shown in Figure 3. The pericarp is formed from several different layers surrounding the endosperm and germ of the kernel. The endosperm and germ form a matrix of carbohydrates, proteins and fats with the germ being higher in fat. Constituent properties of the three major cereal grains, corn, rice and wheat and two legumes are listed in Table 1 where starch makes up from 30 to more than 60 percent while proteins comprise from 5 to 35 percent followed by fat consisting of from 1 to 20 percent. Starch exists as granules within the endosperm while proteins are dispersed as globules within the matrix containing the starch. These constituents are the major polymers in cereals and legumes and play a significant role in water adsorption and retention. Fat and fiber play a relatively insignificant role in water sorption due to their hydrophobic nature and minor constituency.

#### 2.1.1. Starch

Starches are essentially glucose polymers and occur primarily in two forms as amylose and amylopectin. The starch granule consists of a layered structure and contains from 0–70 percent amylose molecules which are generally coiled in their structure and 30–100 percent amylopectin, a molecule with a highly branched structure. The molecular weight, which is related to surface area for water binding, varies in amylose from 4 × 10^4^ to 3.4 × 10^5^ while amylopectin molecular weights range between 4 × 10^6^ to 6 × 10^6^ [101]. There seems to be some confusion concerning the relative water binding capabilities of these two polymers. According to Gallant et al. [85] the crystalline nature of amylopectin in the starch granule prohibits water absorption by the crystalline regions comprised of block lets of amylopectin (Figure 2) compared to the amorphous regions comprised mainly of amylose. The amorphous fraction of starch, where the amylose resides, is suggested to be responsible for starch granule swelling or shrinkage caused by water moving into and out of the lightly branched amylose structure. This indicates that the water binding properties of amylose may be more important than those of amylopectin in granular starch within cereal grains. However, swelling power was found to be negatively correlated to amylose content and positively correlated to longer chain length of amylopectin in wheat starch [102], which contradicts the idea that water absorption by amylopectin is minimal. In either case, the swelling power of starch is related to the water-holding capacity of starch molecules by hydrogen bonding [103].

Starch grains vary considerably in shape from spherical to angular to elliptical shapes as shown in Figure 2. Rounder starch grains tend to have higher amylose content. On average the starch granule has a density of from 1.5 to 1.65 g cm^−3^. Grain size varies from 2 to 175 μm in diameter depending on variety. External specific surface area, *A*_e_, of the starch granule varies considerably. With rice *A*_e_ = 0.8 m^2^ g^−1^ while for potato starch *A*_e_ is approximately 0.085 m^2^ g^−1^ (Table 2). In the case of wheat, examination of 12 different cultivars showed a trimodal distribution in both starch granule size and surface area, with diameters of <2.8, 2.8–9.9 and 9.9–52.7 μm, which contained 51–55, 23–28 and 18–25 percent of the total surface area, respectively [104]. Starch granule external surface area is approximately four orders of magnitude smaller than the internal, macromolecular surface area and is not expected to contribute significantly to water binding by comparison.

The temperature dependence of different starch properties has been studied widely. The thermal conductivity of starch granules was shown to increase with increasing amylopectin content [105,106] and swelling occurred over narrower temperature ranges for pure amylopectin starches of rice and potato when compared to native starches [103,106]. Generally, starch granules swell when placed in water with a volume increase of about 5 percent or to a water mass of about 30 percent of the starch granule dry weight [107]. This swelling is reversible up to a temperature of about 65 °C after which gelatinization takes place and swelling becomes irreversible. Structural changes occurring after gelatinization apparently bind less water as indicated by increased permittivities in gelatinized versus granular starches in solution [39].

**Figure 4:**
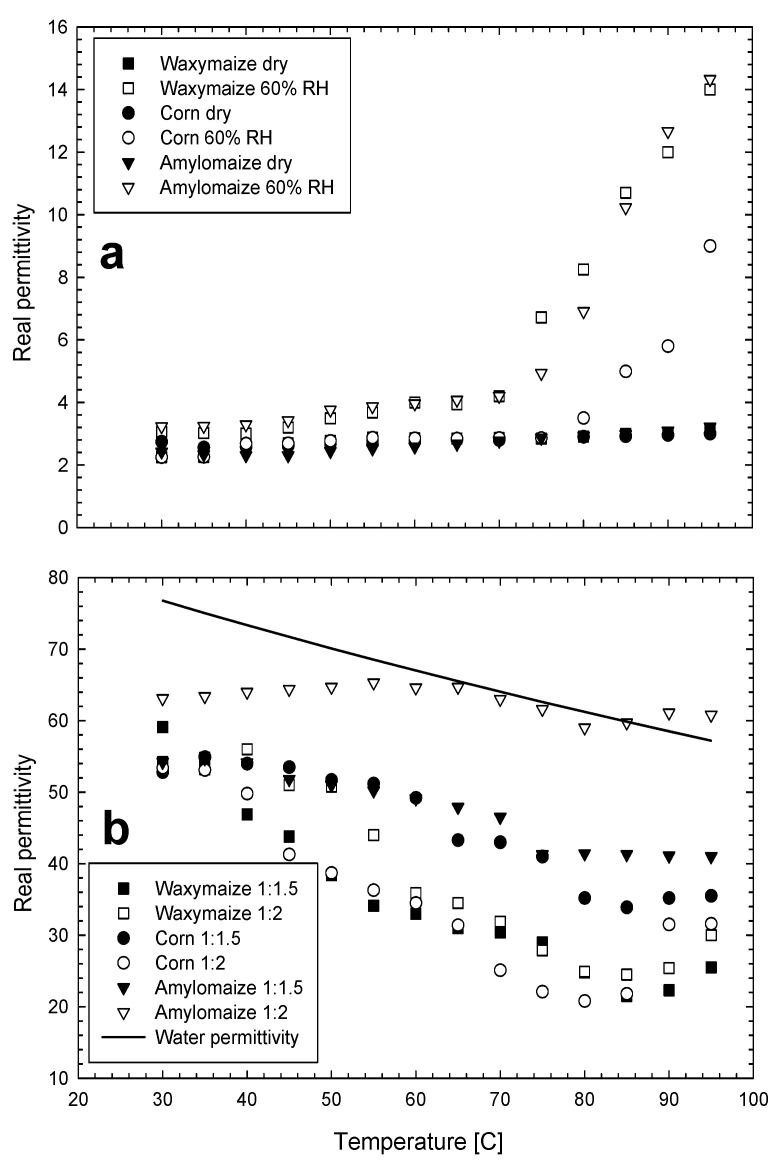
Real permittivity of three different varieties of corn starch at different moisture contents measured at 2.45 GHz (digitized from [73]). Bound water-dominated starch (**a**) shows an increase in the dielectric constant, *ε*’, with increasing temperature while free water-dominated starch (**b**) exhibits a decrease in *ε*’ with temperature increase. Free water permittivity shown for reference.

The effects of temperature on the permittivity of different starches are presented in Figure 4. Ndife et al. [73] demonstrated the opposing relation of temperature on the permittivity of dry and wet starch samples from wheat, rice and three different varieties of corn. Among corn starches, ratios of amylopectin: amylose vary between different varieties such as waxymaize (100:0), maize (70:30), amylomaize (30:70). Starch variety appears to affect the measured permittivity of starch water solutions. Figure 4a illustrates the increase in permittivity with increased temperature for three different varieties of dry and slightly wetted (to 60 percent RH) corn starch. In Figure 4b, the starch water ratios of 1:1.5 and 1:2 illustrate an opposite effect where permittivity decreases with increasing temperature which is, for the most part, explained by the nature of the permittivity of water to decrease with increasing temperature as shown by the solid line in Figure 4b. The temperature-dependent release of bound water (Section 1.2.3) is significant in biological materials as demonstrated by Fukuzaki et al. [66] who found a 25 percent reduction in the bound water of DNA with a temperature increase of from 0 to 25 °C. Accurately modeling this phenomenon would enhance the predictability of moisture content using dielectric measurement techniques.

#### 2.1.2. Proteins

Protein composition ranges from more than 30 percent in legumes to less than 10 percent in cereals. Comprised of twenty different types of amino acids, protein structure varies considerably with coiled, folded and grouped arrangements possible. Protein length may contain between 50 and 500 amino acids. There are 4 classes of seed proteins, which are albumin, globulin, prolamin and glutelin. Most cereal grains contain all four proteins at variable fractions ranging from 5 to 50 percent of each while rice is as high as 80 percent in glutelin. These long chain amino acids connected by peptide bonds have a very large surface area, only a fraction of which is accessible by water. Protein molecules absorb water mainly along their outer surface. The water appears to form a shell around the outside of the molecule with the inside being quite anhydrous [108]. Assuming a monolayer of bound water surrounding the protein, Fisher [109] found the limiting hydration content (monomolecular layer) to be almost constant (0.28 ± 0.02 g H_2_O/g protein) for 34 different proteins studied. Kuhn et al. [110] analyzed the surface of 56 different protein structures and found surface areas ranging from 880 to 37,329 Å^2^ with a mean value of 7495 ± 5981 Å^2^. They suggested that bound water molecules prefer groove to no groove surfaces of the protein by more than three times and grooves accounted for 25 percent of the total surface area while binding 50 percent of the water molecules. The molecular weight of proteins is well correlated to various aspects related to water absorption and dielectric measurement. Richards [111] gave an approximation for protein accessible surface area, *A*_a_ (Å^2^), based on molecular weight, MW, given as:

(12) $$A_{a}=11.12\cdot MW^{\frac{2}{3}}\;$$

Molecular weights vary widely among the four plant proteins and even among species with albumins and prolamins having substantially lower molecular weights than globulins and glutelins. Using protein fraction, f_i_, and molecular weight of each of the four cereal protein molecules, we estimated specific surface area, *A*_s_ (m^2^ g^−1^), for different cereal grains shown in Table 3. The calculation is based on the protein molecular mass, *M_i_* (g molecule^−1^), which is given as:

(13) $$A_{s}=\overset{4}{\underset{i=1}{\sum }}\frac{A_{a}f_{i}}{M_{i}}\;$$

The calculated specific surface areas of the proteins range from 1000 to 2000 m^2^ g^−1^ while their contribution relative to the total kernel mass ranges from 100 to 300 m^2^ g^−1^.

**Figure 5:**
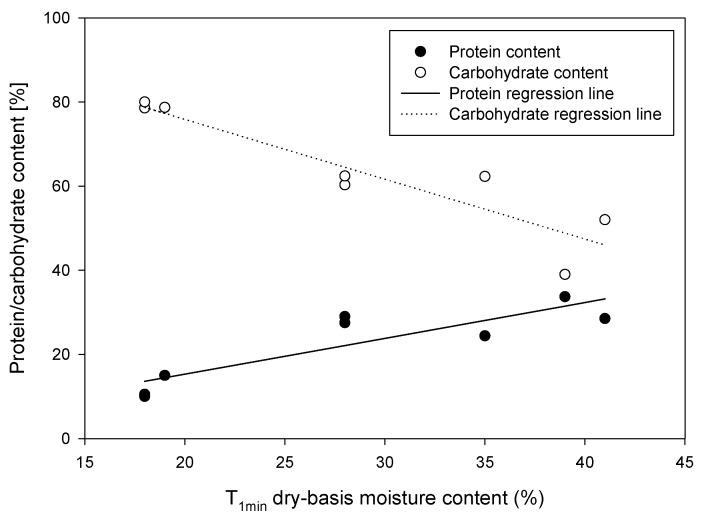
Protein and carbohydrate content of 8 different cereals and legumes versus the dry-basis moisture content at T_1min_. Authors Ratkovic and Pissis suggested the water content corresponds to the primary hydration sphere around the macromolecule (digitized from [113]).

Even though starch is the dominant constituent of cereal grains, the second major component, protein, seems to play a similar role in terms of water absorption and water binding [112]. There appears to be a phase change which occurs just below 20 °C in some legumes which Leopold [112] suggests is related to characteristics of the bulk constituents (i.e., lipids, proteins). Ratkovic and Pissis [113] showed a high correlation of both protein and carbohydrate content in various cereals and legumes to the water content at *T*_1min_ (Figure 5) using nuclear magnetic resonance (NMR). *T*_1min_ refers to the water content corresponding to the primary hydration sphere around the macromolecule. This correlation is associated with the monolayer water surrounding the macromolecules where the *T*_1min_ values fell between 25 to 30 percent of the maximum hydration for all grains studied. Using the thermally stimulated depolarization currents (TSDC) method, they determined moisture contents for bound water in the primary hydration layer to be 0.18 g g^−1^ (db) for beans, pea and chick pea, and 0.10 g g^−1^ for wheat. Using additional information on soluble carbohydrates provided in Ratkovic and Pissis [113], we fitted *T*_1min_ data as a function of both protein (P) and carbohydrate (C) contents in percent giving the following relation for the monolayer moisture content (*M*_ml_) on a dry weight basis as:

(14) $$M_{\mathrm{ml}}=exp(0.612-3.06\times 10^{-5}P^{3}-4.03\times 10^{-5}C^{2.5})\;$$

The dielectric constants of pure (dry) proteins vary considerably depending on protein type. Loffler et al. [118] found a static dielectric constant of 15 for a zinc peptide and calculated the static dielectric constant of water associated with the protein to be 47. In reviewing previous work, he referenced protein dielectric constants of from 2 to 5 where side chain interactions were neglected and when included, dielectric constants ranged from 16 to 37 depending on the modeling approach. Sham et al. [119] suggested that the actual value of the protein’s internal dielectric constant is not well related to the macroscopic protein dielectric constant and that this value should be 4 or higher. Simonson and Perahia [120] suggest that the dielectric constant of a protein is not unique and difficult to define using various methods including measurement. Values based on calculations for Cytochrome C range from 2–5 while intermediate values on the order of 10 have been determined for the active site of trypsin and even larger values (20–35) are found for charged mobile side chains. Values of 4–8 are consistent with measurements in Cytochrome C as well as predictions based on their simulations. Suzuki et al. [121] used a value of 2.5 for modeling the frequency spectrum of various proteins. Gilson et al. [122] who modeled the electrostatic interactions in proteins, point out that the actual dielectric is a function of many complicating factors including protein shape and orientation with respect to the background, solvent and the applied electric field. Calculated permittivity based on electronic and atomic polarization results in a permittivity of 2 [123], which is considered by some to be the dielectric constant of proteins at low temperatures and high frequencies.

Proteins appear to play a significant role in permittivity measurements of cereal grains from which water content is inferred. The variety of grain or legume dictates the type and quantity of protein which is related to the protein water binding surface area. Estimates of surface area based on the molecular weight of each protein type and their constituent makeup have been used previously. Combining these predictions of protein water binding capacity with estimates for starch water binding should enhance modeling of the bound portion of water within the grain or legume. In the absence of detailed information on surface area or bound water content, Equation (13) provides estimates of monolayer water using only protein and carbohydrate content.

#### 2.1.3. Fat and Cellulose

Holmes et al. [65] examined the effect of seed coat on the complex permittivity of various varieties of grains. Both dielectric constant and loss factor were increased in some cases and decreased in others as functions of water content of the seed in response to the presence of the seed coat. In particular, the thick, moisture-absorbent seed coat of one variety in particular (*Cucurbita pepo*) was responsible for substantial increases in the dielectric constant at high moisture contents. By comparison, the thin seed coat of corn (*Zea mays*) had little effect on the permittivity measurements throughout the moisture content ranges measured. The relatively low permittivity of lipids and cellulose, along with their sometimes hydrophobic and low water-absorption capacity, limits their importance in permittivity measurements for water content. However, knowledge of the content of oil and cellulose in the grain may indicate the extent to which water binding is reduced, especially for the case of high-oil content seeds and legumes.

### 2.2. Density and Porosity of Packs of Different Agricultural Grains

Next to water content, grain size, shape and packing are the most critical factors affecting the permittivity measurement. Grain bulk density or porosity determines the kernel volumetric presence and is water content-dependent. Therefore, the determination of grain packing is coupled to water content determination. The physical characteristics of cereal grains such as kernel shape and kernel density produce variations in bulk densities and porosities as shown in Table 4. Density and porosity may be viewed at different scales and we wish to distinguish between bulk and kernel characteristics. Bulk density, *ρ*_b_ (g cm^−3^), and bulk porosity, *ϕ*_b_, depend on the volume packing of kernels, as opposed to kernel density, *ρ*_k_ (g cm^−3^), and kernel porosity, *ϕ*_k_, which refer to individual kernels. We also refer to “solid” density, *ρ*_s_ (g cm^−3^), which specifically references the molecular density of the solid portion of a kernel or starch grain (i.e., excluding void volume between solids).

Kernel shape and density directly impact the packing, arrangement and ultimately *ρ*_b_ and *ϕ*_b_ of the grain. Both *ϕ*_k_ and *ϕ*_b_ are critical factors in cereal grain permittivity measurement for water content determination, where each is generally given on a wet weight basis. The kernel density is a function of the moisture content and directly influences the bulk density. As will be demonstrated here, kernel shape can also have a significant influence on the bulk density. The bulk porosity of the grain, *ϕ*_b_, can be described as a function of *ρ*_k_ and *ρ*_b_ through the following relationship.

(15) $$\phi_{b}=1-\frac{\rho_{b}}{\rho_{k}}\;$$

Bulk porosity is an important factor in the drying and storage of cereal grains, especially where heated air is passed through the grain for drying. Knowledge of the bulk porosity of cereal grain is important for designing aerodynamically efficient drying systems. We will present several physical descriptors for predicting the kernel and bulk density relative to moisture content and kernel shape.

#### 2.2.1. Kernel Density

The bulk density of grain (i.e., pack of kernels) is related to the bulk pack porosity through the individual kernel density. A straightforward approach to computing kernel density is to sum the constituents of the kernel (see Table 1) multiplied by their respective densities as:

(16) $$\rho_{k}=\frac{1}{\sum_{i=1}^{n}\left(\frac{x_{i}}{\rho_{i}}\right)}\;$$

where *x*_i_ is the mass fraction and *ρ*_i_ is the constituent density. Peleg [125] gives densities of common constituents of grains such as starch (1.5 g cm^−3^), protein (1.4 g cm^−3^), cellulose (1.27–1.61 g cm^−3^) and fat (0.90–0.95 g cm^−3^). Such detailed information on constituent quantities, however, is often not available and other approaches must be employed. Since kernel density is a function of moisture content, we compute *ρ*_k_ based on the mass and volume of solids (*m*_s_, *V*_s_) and water (*m*_w_, *V*_w_) contained within the dynamic volume of the kernel, given as:

(17) $$\rho_{k}=\frac{m_{s}+m_{w}}{V_{s}+V_{w}}\;$$

**Figure 6:**
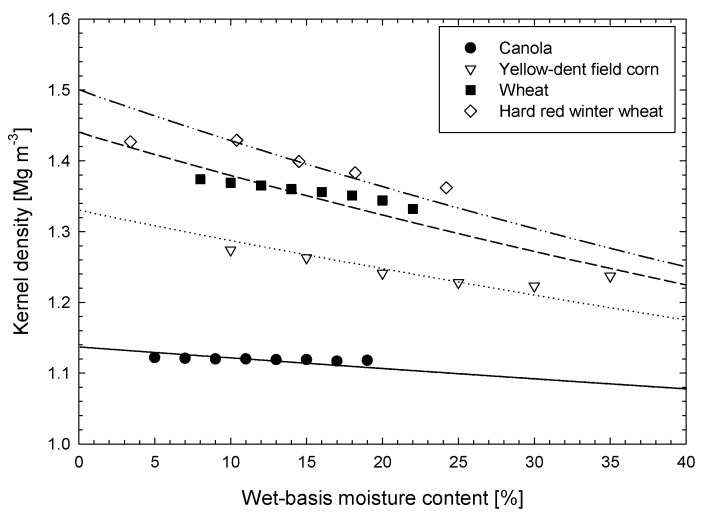
Measured kernel densities of canola, wheat, hard red winter wheat (all digitized from [126]) and corn (digitized from [127]) compared to modeled density using Equation (17) in terms of wet-basis moisture content.

For this relationship, Sokhansanj and Lang [126] describe *ρ*_k_ as a function of the density of the dry kernel solids, *ρ*_s_, and the moisture content as:

(18) $$\rho_{k}=\frac{\rho_{s}}{1+\left(\frac{\rho_{s}}{\rho_{w}}-1\right)M_{\mathrm{wb}}}\;$$

where *ρ*_w_ is the density of water (e.g., 1 g cm^−3^) and the moisture content on a wet-basis is *M*_wb_, which is equal to *m*_w_/(*m*_s_ + *m*_w_). We compared this relationship to measured kernel densities for canola, wheat and corn shown in Figure 6. As the kernel dries, the tendency of the measured kernel density to increase at a slower rate than the physical model, may arise from the gradual displacement of water within the kernel with air (desorption) or of kernel air voids with water (sorption), depending on the process.

The dry kernel density values given in Figure 6 were obtained by fitting each line to the measured data of Nelson [127] for corn and data for wheat and canola were taken from Sohkansanj and Lang [126]. The fitted kernel densities do not account for voids which develop in drying kernels at moisture contents below storage values (e.g., 13 percent). Referring to voids within grain constituents such as protein, the specific volume of the dry protein crystal was constant from a weight fraction of water of from 0 to 0.1 where the voids between albumin molecules had not completely filled with water [111]. The range of inter-protein molecule porosity has been measured in the range of 0.3 to 0.7 and is generally between 0.4 to 0.6. Chang [128] found kernel porosities of corn, wheat and sorghum to range from 0.03 to 0.13 at storage moisture contents between 0.11 and 0.14 on a wet-basis. Chang [128] also found that more than 80 percent of the internal pore space of corn kernels were inaccessible by Helium gas used in density measurements, suggesting difficulty in obtaining accurate dry kernel density measurements using such methods. Although dry kernel densities are more difficult to measure and are subject to errors from undetectable voids, cereals and legumes are rarely dried below storage moisture contents of 10 to 15 percent and from this point up to harvest moisture levels, modeled and measured densities are somewhat linear and in good agreement.

For grains harvested at relatively high moisture contents such as corn for seed, the typically measured kernel density tends to be greater than that predicted by the model. This may be partially explained by the fact that while attached to the plant, the developing kernel maintains turgor pressures from 0.2 to 0.3 MPa [129], which may cause densification of various kernel constituents during seed maturation. These constituents may expand, unfold and relax during kernel desiccation, with little change in kernel volume, leading to a reduction in the overall kernel density. The process of kernel desiccation is accompanied by a change in kernel shape and volume. Leopold [112] demonstrated volume increases of soybeans with hydration which were substantially greater than the weight of water imbibed. He suggests that polymeric seed constituents unfold with hydration which we suggest is a mechanism for increased surface area for water binding, discussed in Section 2.3.1. This physical relationship is important in determining the packing of grains and may be used for improving predictions of bulk density.

#### 2.2.2. Grain Constituent Arrangement and Shape

The structural hierarchy of cereal grains is complex with many levels where the constituent shape is of a general form and likeness (Figure 2). For many cereal grain constituents, such as the kernel or starch grain, shape approximations using prolate (e.g., rice, wheat, barley) or oblate (e.g., lentils) shapes are appropriate [130]. Volume and surface area of spheroids (also ellipsoids) are readily calculated. For spheroidal packings such as lentils, barley, wheat, etc., an approximation of bulk porosity, *φ*_b_, uses the ratio of the kernel volume to surface area [131]:

(19) $$\phi_{b}=1-k\frac{V_{s}^{\frac{2}{3}}}{S_{a}}\;$$

where *k* is an empirical fitting coefficient, vs. is the volume of the spheroid and *S_a_* is the spheroid surface area. A single empirical expression for the unique $\frac{V_{s}^{\frac{2}{3}}}{S_{a}}$ relation of the spheroidal geometry was fitted (*R*^2^ = 0.999) to computed values for aspect ratios ($\frac{a}{b}$. ) from 0.1 to 10, giving the following relation:

(20) $$\frac{V_{s}^{\frac{2}{3}}}{S_{a}}={\left[-2.09+3.49\cdot \ln\left(\frac{a}{b}\right)+6.92{\left(\frac{a}{b}\right)}^{-\frac{1}{2}}\right]}^{-1}\;$$

**Figure 7:**
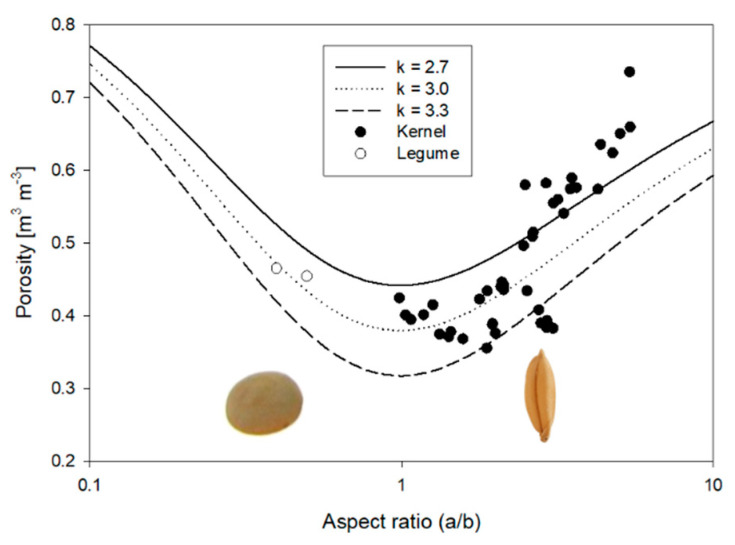
Bulk porosities in various legume (*a* < *b*) (digitized from [132]) and grain kernel (*a* > *b*) (digitized from [130]) packings as a function of legume or kernel aspect ratio. Modeled porosities are shown for a range of scaling coefficients, k, using Equations (19) and (20).

Porosities derived from measured kernel and bulk densities [130,132] of different seeds, cereal grains and legumes ranging in variety, all at relatively dry moisture contents (5–9 percent, wb), are plotted as a function of aspect ratio in Figure 7. Modeled porosities as functions of aspect ratio are plotted using Equations (19) and (20) using three different scaling coefficients for comparison. The volume—surface-area relationship reproduces the general trend of increased porosity as aspect ratio deviates from that of a sphere (*a*/*b* = 1). However, the fitting parameter is incapable of improving the fit to data beyond vertically scaling the curve. Jones and Friedman [11] found a good correlation of this model in porosity data of different solid materials covering a much larger aspect ratio range of from 0.04 to 50.

#### 2.2.3. The Grain Bulk Density

A prediction of the bulk density of grain packing based on their physical characteristics is of value for various aspects of harvesting, processing and storage. Using the information on kernel density coupled with shape-based porosity predictions described previously, we obtain a general prediction of bulk density as a function of moisture content using Equations (14) and (17)–(19). The resulting expression is given in terms of dry kernel density, moisture content and aspect ratio as:

(21) $$\rho_{b}=\frac{k\rho_{s}}{\left[-2.09+3.49\cdot ln\left(\frac{a}{b}\right)+6.92{\left(\frac{a}{b}\right)}^{-\frac{1}{2}}\right]\cdot \left[1+\frac{\rho_{s}}{\rho_{w}}-1M_{\mathrm{wb}}\right]}\;$$

**Figure 8:**
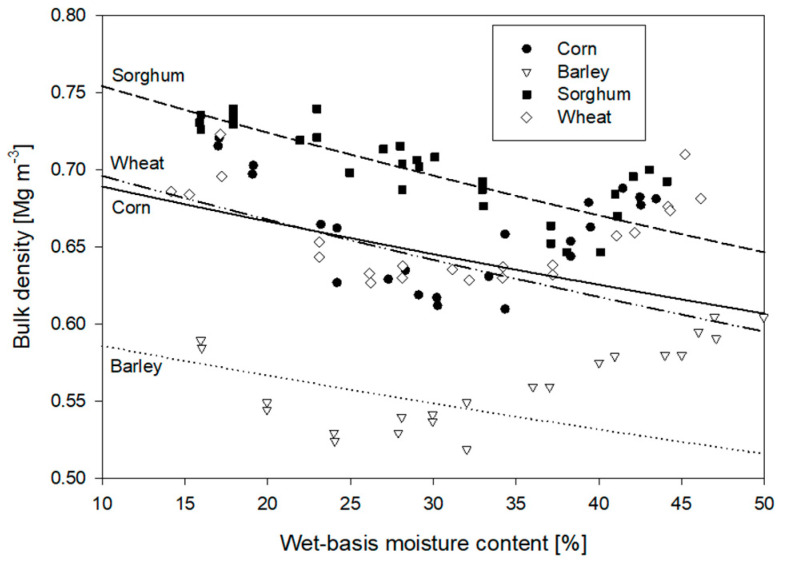
Measured bulk densities of four different cereals (digitized from [133]) compared to modeled results using Equations (7), (9) and (10) plotted as a function of wet-basis moisture content.

These estimates are compared to bulk densities of four different cereals measured by Brusewitz [133] shown in Figure 8. The modeled result correlates best to bulk densities at mid-range water contents where many cereals are harvested. Poorer correlation is noted at higher moisture contents, which may be a combined result of the increase in kernel density seen in Figure 6 and the alteration in kernel shape which tends to be more rounded and softer (i.e., leading to deformation and reduced bulk porosities) at higher moisture values.

Advances in measurement technology continue to provide more accurate moisture content measurements and to improve the potential for accurate bulk porosity and density measurements as well. Using radio-frequency or microwave attenuation and phase spectra [134,135] combined with temperature data [136] density-independent measurements of cereal grain moisture content have been achieved [137,138]. Unfortunately, such measurements largely remain in the research realm, requiring expensive equipment, highly skilled operators and grain variety-dependent calibration coefficients.

#### 2.2.4. Frequency-Domain Measurements

Permittivity measurements made in the frequency domain contain richer information content than in the time domain. There are several features of the spectra that can be useful in correlating measurements to physical phenomena related to relaxation effects arising from external factors of temperature, water content, electrical conductivity and other physical properties and processes. An example of such physically-based phenomena has been identified in the temperature-dependent real permittivity of a variety of porous materials, including soil, whey, fruits and vegetables.

**Figure 9:**
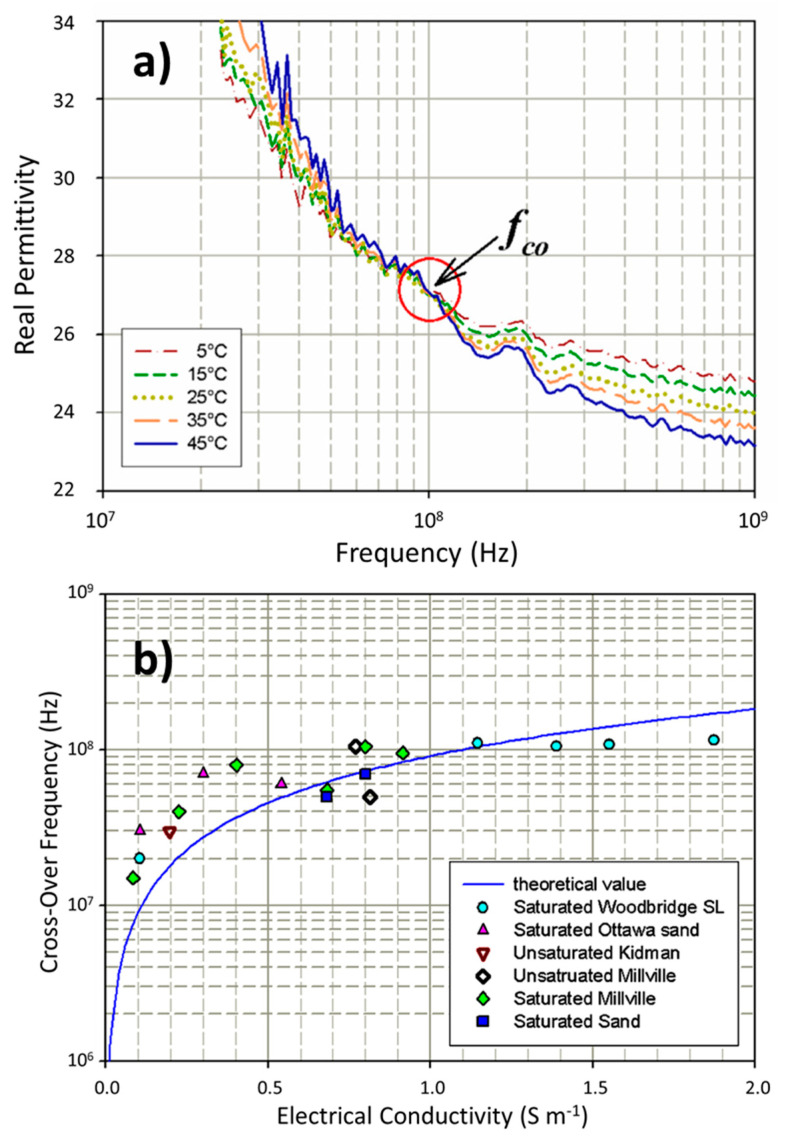
(**a**) Cross-over frequency illustrated in the frequency-dependent real permittivity spectra of Woodbridge silt loam at a water content of 0.15 m^3^ m^−3^ and solution electrical conductivity (EC) of 0.9 S/m measured at different temperatures (modified from Chen and Or [139] Figure 7b). (**b**) Cross-over frequencies determined as in (**a**) for different soils with the modeled values as functions of electrical conductivity of the bulk soil (modified from Chen and Or [139] Figure 9).

This phenomenon illustrated in Figure 9a, was identified by Wraith and Or [46] and termed the crossover frequency by others [139,140] where the real permittivity spectra at different temperatures all coalesce to a single permittivity value, below which, higher temperature samples exhibit higher real permittivity and above which, the lower temperature sample permittivities are higher instead. Chen and Or [139] plotted cross-over frequencies of different soils as functions of their corresponding bulk electrical conductivity (Figure 9b), demonstrating a reasonable fit to a physically-based model suggesting the cross over frequency increases with increasing electrical conductivity between 10 and 100 MHz.

Table 5 lists a variety of porous materials and their corresponding crossover frequencies and temperature ranges over which measurements were made. Wraith and Or suggested that the time domain reflectometer (TDR)-measured “real” permittivity was determined by an interplay between competing phenomena of (1) the reduction in bulk water apparent or real permittivity with increasing temperature and (2) the increase in real permittivity with increasing temperature due to a transition of bound water to free water. Nelson [140] suggested that at frequencies below this crossover frequency, ionic conduction was dominating the real permittivity, but above that point dipolar relaxation became the dominant control. Obviously, there are complex and potentially confounding processes at work within porous media that are affected by temperature. Further probing of these phenomena within the frequency spectra of porous media should uncover additional details and potential mechanisms at play.

### 2.3. How Bound Water Content Affects Permittivity

Water molecules become hydrogen bonded to polymers within cereal grains resulting from the relative numbers of polar and non-polar groups and their steric accessibility within molecules. Based on these concepts, proteins and carbohydrates containing many polar sites, have the highest affinity for water, while lipids or hydrocarbon chains consisting of high proportions of non-polar groups are hydrophobic. Not all polar sites are capable of binding water either. In starch for example, only hydroxyls can bind water for steric reasons, while in proteins only polar sites of the main chain and hydrophilic amino acid chains bind water. In the case of lipids, only the extremity of the long apolar aliphatic chains can link water and therefore very little water is attached to lipids. Ultimately, the binding of water molecules to solid surfaces alters the permittivity of the water and thus the overall measurement result.

#### 2.3.1. Water Binding in Proteins, Starches and Flours

The various levels of water binding in constituents of cereal grains are generally described as either monolayer or multilayered, the latter of which is thought to consist of 2 to 3 layers (Table 6). Values of both monolayer and multilayered bound water in a variety of kernel constituents and whole grains are presented on a dry weight basis in Table 6. Methods used for determining bound water content in these studies are listed, which likely add to some of the variation in determined values of bound water among the different methods. The monolayered water content has been associated with T_1min_ and short T_2_ spin lattice relaxation times associated with NMR techniques. Relaxation times in corn starch were shown to increase with increasing moisture content [143,144]. Botlan et al. [143] found it surprising that the quantity of bound water (monolayered) increased regularly over the full range of water contents studied (0.14 to 0.94 g g^−1^) even when a large amount of “weakly bound” water is present. This suggests that perhaps the monolayered bound water fraction increases with increasing moisture content as a result of increasing surface area. This may arise from swelling and increased solubilization of the amorphous regions of the starch grain comprised of amylose and amylopectin polymers (Figure 3d). During the drying process, the quantity of monolayered bound water is diminished initially by the reduction in turgor pressure and associated water loss.

Shrinkage, folding and rearrangement of tissues, result in continued reduction in kernel volume (e.g., lentil volume reduced 17 percent from 24 to 5 percent moisture content [153]) and in reduced accessible internal surface area. The source of this “dynamic surface area” may occur within the highly branched amylopectin clusters (Figure 3d) of starch or within the amorphous regions of the kernel containing proteins and is likely a function of polymer type as well as moisture level [144]. This phenomenon may explain the irregular nature of the permittivity versus water content relationship of starch which exhibits a reduced rate of permittivity rise with increasing moisture content near mid-range moisture levels [26]. Other temperature related phenomena have been noted. Leopold [112] showed a shift in slope of the otherwise linear seed volume—temperature relationship of soybeans just below 20 °C, which suggests a phase change, which may be due to lipid or protein constituents within the seed. Such behaviors pose further challenges for modeling the bound water component of the permittivity/moisture content relationship and require more detailed information concerning the surface area evolution and other mechanisms acting at a molecular level.

One method of obtaining an estimate of the surface area of the kernel or starch grain is derived from the measured monolayered water content (Table 6) and an estimate of the density of the monolayered bound water. The assumed structure of the water molecules will also influence the estimate slightly. Ryden [154] suggested a relationship for water molecule spacing, *s* (Å), based on water density, *ρ*_w_ (g cm^−3^) and packing given by:

(22) $$s=\sqrt[{3}]{\frac{K_{p}}{\rho_{w}}}\;$$

where *K*_p_ is a packing constant given as 28.21 (g Å^3^ cm^−3^) for tetrahedral and 29.92 (g Å^3^ cm^−3^) for cubic packings. Assuming a water density of 1 g cm^−3^, the resulting water molecular spacings are 3.04 Å and 3.10 Å for tetrahedral and cubic packing, respectively. These values are representative of intermolecular spacings of free water [155]. For bound water, however, two contrasting views have been presented. One suggests that the density of bound water is less than the density of free water [156,157] leading to larger spacings between water molecules. Reduced densities were suggested to arise from inter lattice spacing constraints between clay platelets which were numerically simulated, producing fluid densities both greater than and less than free water density depending on platelet separation [158]. For cereal grain constituents of protein and carbohydrate molecules, it is also conceivable that for certain structural spacings within and between molecules, reduced water densities are achievable.

Another view suggests water densities greater than that of free water [56,159], which corresponds to reduced water molecular spacings. Gur-Arieh et al. [56] showed a constant wet flour density for moisture contents (wb) from 0 to 0.07 g g^−1^, after which the wet density decreased steadily up to the final measured moisture content of 0.26. From this result they calculated a monolayer water density, *ρ*_ml_, of 1.48 g cm^−3^ and a density of the second molecular layer of water to be 1.11 g cm^−3^. The remaining water layers were calculated to have a density of 0.967 g cm^−3^. For a tetrahedral packing and using a monolayer water density of 1.48 g cm^−3^ and a second layer density of 1.11 g cm^−3^, Equation (15) gives molecular spacings of 2.67 and 2.94 Å, respectively. These estimates lie on either side of spacings found in ice (hexagonal) of 2.76 Å [155] and are similar to other estimates of bound water spacings of between 2.5 Å and 2.8 Å [160]. For monolayered water content given on a dry-basis, *M*_ml_, an estimate of the specific surface area, *A*_s_ (m^2^ g^−1^), is given using the estimated monolayer water density, *ρ*_ml_ (g cm^−3^), and packing constant, *K*_p_ (Equation (21)), given by:

(23) $$A_{s}=\frac{M_{\mathrm{ml}}}{\rho_{\mathrm{ml}}^{\frac{2}{3}}\cdot K_{p}^{\frac{1}{3}}}\;$$

Using the tetrahedral packing assumption with the associated density and spacing mentioned above, we computed *A*_s_ for different proteins, starches and grains shown in Table 7, by multiplying the dry-basis water content measurements for monolayered water by the constant 2531 m^2^ g^−1^ (reciprocal of denominator in Equation (22)). Specific surface area estimates for pure proteins average 600 m^2^ g^−1^, while starch specific surface area averages 400 m^2^ g^−1^. Further evidence of higher surface area associated with protein content was given by Konsta et al. [152] who found the completion of the primary hydration layer in wheat and bean flour to be 0.1 and 0.15 g g^−1^ wb (0.11 and 0.17 g g^−1^ db), respectively. Higher protein content in bean flour, which presents more hydration (polar) sites than do carbohydrates, was given as the reason that beans bind larger amounts of water than wheat. Specific surface area estimates shown for bean and soy flours in Table 7 are similar in magnitude to measurements in the highest surface area clays (i.e., 800 m^2^ g^−1^ [161]).

The internal water accessible surface area of the solid is an indispensable parameter used in modeling the bound water permittivity of the liquid phase in porous media. Using constituent geometries of cereal grains, calculated surface areas at different scales provide insight into the approximate level to which water is absorbed by the solids. Table 7 shows computed specific surface areas for structural components of corn starch. Comparison of calculated *A*_s_ values to those shown in Table 5 indicate that water is absorbed well into the amylopectin clusters and the surrounding amorphous regions (see Figure 2) where cumulative values of *A*_s_ approach 500 m^2^ g^−1^.

#### 2.3.2. Modeling Grain Constituent Water-Phase Permittivity

**Figure 10:**
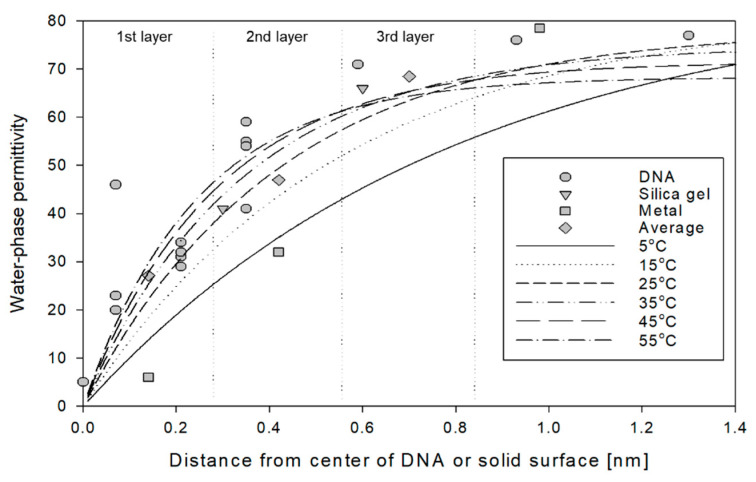
Measured and modeled water-phase permittivity as a function of distance from the center of DNA [162] or distance from the surface of silica gel [53] and metal [163] (digitized data). Computed temperature dependent permittivities based on free water permittivity and on bound water radius (*r* = 2.5 × 10^−10^), specific surface area (*A*_s_ = 400 m^2^ g^−1^) and relaxation frequency (f* = 1 GHz) from Equation (22).

The large permittivity contrast between air (*ε*_a_ = 1) and free water (*ε*_fw_ = 80) provide high resolution moisture content measurements at high water contents, whereas the small contrast among air, bound water and biological tissue gives lower resolution to measurements at low water contents. Water binding to solid surfaces reduces the mobility of the water molecule and the result is a reduction in the water phase permittivity. This phenomenon has been widely studied. Lamm and Pack [162] modeled localized permittivities in water-saturated DNA molecules using the Poisson-Bolzmann approach. Local permittivities were calculated in major and minor grooves and elsewhere, and average permittivity was computed as a function of distance from the center of the DNA molecule (Figure 10). The relation of increased permittivity with distance from the center of the DNA molecule corresponds closely to that found by Thorp [53], who measured the permittivity of the first and second molecular layers of water on silica gel also shown in Figure 10. Bockris et al. [163] suggested water-phase permittivity values for the first and second monomolecular layers of 6 and 32, respectively. Using an average water layer spacing of 0.28 nm [164], we plotted the arithmetic average permittivity of each layer using the previously discussed data, shown in Figure 10.

Models have been developed which represent the water-phase permittivity as a function of distance from the surface of soil particles based on surface area and bulk density [59,165] or individual particle size and shape [11]. Jones and Or [26] combined the temperature-dependent permittivity of bound and free water into a single expression reducing the number of water phases from 2 to 1. An exponential growth function provided the approximate form for modeling measured and calculated permittivity data near solid surfaces. The bound water temperature-dependent expression, *x*(*T*), from Equation (4) (*r* = 0.25 nm, *f* = 1 × 10^−9^ Hz, [59] was coupled with Equation (2), describing the temperature dependence of the permittivity of free water, *ε*_fw_(*T*), resulting in the following:

(24) $$\varepsilon_{w}\left(T\right)=\varepsilon_{\mathrm{fw}}\left(T\right)\left[1-exp\left(\frac{-t_{w}}{x\left(T\right)}\right)\right]\;$$

where *t*_w_ (m) is the water film thickness equal to *M*_v_/*A*_s_*ρ*_b_ and where *A*_s_ is known or estimated from methods described herein (e.g., Equation (22) and Table 5). This expression is plotted in Figure 10 as a function of temperature and distance from the solid surface. The average of the measured and simulated permittivities for monomolecular layers 1, 2 and 3, designated in Figure 10, are 27, 47 and 69 while corresponding modeled (*T* = 25 °C) values are 22, 49 and 63, respectively. For a temperature increase, this function shows both the tendency of the water phase permittivity of the bound water fraction to increase, and demonstrates the free water permittivity (greater than 3 layers) decrease with increasing temperature.

To summarize this section on grain physical properties, we point to three key points. (1) The starch and protein composition of cereal and legume grains plays a significant role in the water binding nature of the grain and the water accessible surface area can be calculated based on structural dimensions for starch and on molecular weight and content of the four classes of plant protein. (2) Kernel density and packing characteristics impact permittivity measurements and expressions describing kernel density as a function of water content and shape-dependent porosity may be combined to estimate grain bulk density. (3) The water binding capacity of a porous medium is related to the water accessible specific surface area, which may be estimated from measured mono- and multi-layered bound water determinations. An expression for estimating the temperature-dependent water-phase permittivity, which transitions from low-dielectric bound water to high-dielectric free water over the first 3 to 4 monolayers of water, were described.

## 3. Sample Hierarchical Modeling of Permittivity

In previous sections we have discussed factors influencing permittivity measurements of agricultural grains and their constituency. There are few modeling efforts which address more than one or two of these factors simultaneously. A goal of future work is to include these factors in a comprehensive modeling framework for predicting permittivity (and ultimately moisture content). One of the limitations for such work is the lack of a complete data set which would facilitate an in-depth examination of these factors and their respective influence. It also arises out of the difficulty of isolating an individual effect in the midst of other confounding factors.

Dielectric mixture models use knowledge of constituent permittivities and volumetric fractions of major components (i.e., water, air, solid) to model the permittivity of mixtures. The information requirement for mixture model calculations presents a drawback since constituent volume fractions are not readily determined in packings of cereals and legumes without making concurrent and independent measurements of bulk density and water content. Estimates of kernel and bulk porosities and densities based on grain shape and information on water binding, facilitate approximations of constitutive volume fractions and water-phase permittivities. Inverse bounds on constituent volume fractions derived from complex permittivity measurements and evaluation of the structural moments and geometry [166] may provide improved measurement capability where bulk density or porosity are often both an unknown and confounding factor for cereal grain water content determination.

In harvested cereal grains, water within the grain is a combination of bound and free water while at storage water contents (e.g., 13 percent wb), water appears to be almost entirely bound within the carbohydrate-protein matrix. As water is added to this matrix during hydration, it is absorbed in the macromolecule phase until near saturation, there is a phase separation into fully hydrated macromolecules and free water. The interface of the free water and starch/protein molecule forms a fuzzy line made up of bound water whose properties are a combination of the two adjacent phases [167]. Such a system has been modeled using a spherical macromolecule surrounded by a water phase shell [168,169]. In the following section we will apply this concept to cereal grains for a simple 2-phase case by volume averaging the permittivity of the solid and air phases forming the background phase and using inclusions of water whose shape is that of the whole grain.

### 3.1. Two-Phase Mixtures

The kernel-air packing of grains has been described using the simplest case of a two-phase mixture approach. Nelson [88] applied six different two-phase dielectric mixture equations to estimate the permittivities of wheat and rice kernels from permittivity measurements on bulk grain samples near the storage moisture content. A drawback to applying two-phase mixture theories to cereal grains and legumes is in how to approximate the complex 3 or 4 phase system of air, solid and bound- and free-water as only two phases. The kernel’s solid-water constituency is the source of large variations in permittivity and bulk density. Numerous approaches for reducing the various phases to a two-phase system can be imagined. Arithmetically averaging the air and solid phase permittivities (these being similar in magnitude) and summing their volumetric fractions, provides a rough approximation of the background phase inside of which the water phase is introduced as inclusions. The water phase may be modeled using Equation (23) which accounts for both bound and free water based on surface area estimates.

**Figure 11:**
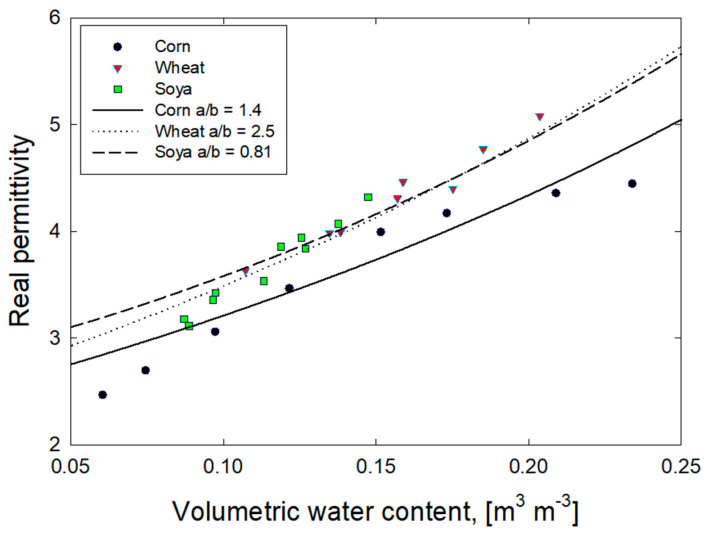
Measured real permittivities in corn, wheat and soya (digitized from [12]) with model predictions using aspect ratios shown and other parameters listed in Table 8.

For a modeling exercise, we selected measured dielectric constant data for corn, wheat and soya measured at 200 MHz [12], which placed emphasis on high moisture corn. Measured real permittivities for “settled” corn, wheat and soya were modeled using the two-phase Maxwell-Garnett mixture theory (Equation (7)). Parameters used in the modeling are listed in Table 8 and the water content-dependent bulk density was computed with Equation (20) with *k* = 3. Solid phase permittivities for corn (3.3), wheat (3.8) and soya (4) were individually adjusted to provide the best fit to data. The resulting permittivity versus volumetric water content predictions are plotted in Figure 11 for grain aspect ratios of 1.4 (corn), 2.5 (wheat) and 0.81 (soya). The resulting model predictions are within reasonable agreement for wheat and soya, which exhibit a uniform monotonic increase in permittivity with moisture content, but corn data show reduced permittivities occurring over a much wider water content range. Additional modeling capacity, which is provided by a three-phase mixture model, should be useful for modeling the more complex permittivity-water content relationship of the corn.

### 3.2. Three-Phase Mixtures

Modeling the permittivity of mixtures, which account for more than two phases requires certain simplifying assumptions regarding the geometry and configuration to facilitate mathematical derivation. The assumption of a spheroidal geometry was used to approximate the kernel shape or other constituents which are complex and often varied, geometrically, spatially and temporally. The designation of phases within a concentric spheroidal configuration is given from inside to outside as core-shell-background corresponding to, for example, solid-water-air (SWA). Permittivity values used for the solid, water and air phases are listed in Table 8, where the permittivity of water is computed from Equation (23) at 25 °C. The calculation of volumetric fractions based on porosity, water content and spheroid dimensions are given in Table 9 for three different phase configurations.

#### 3.2.1. Shape, Surface Area and Phase Configuration Effects on Permittivity

**Figure 12:**
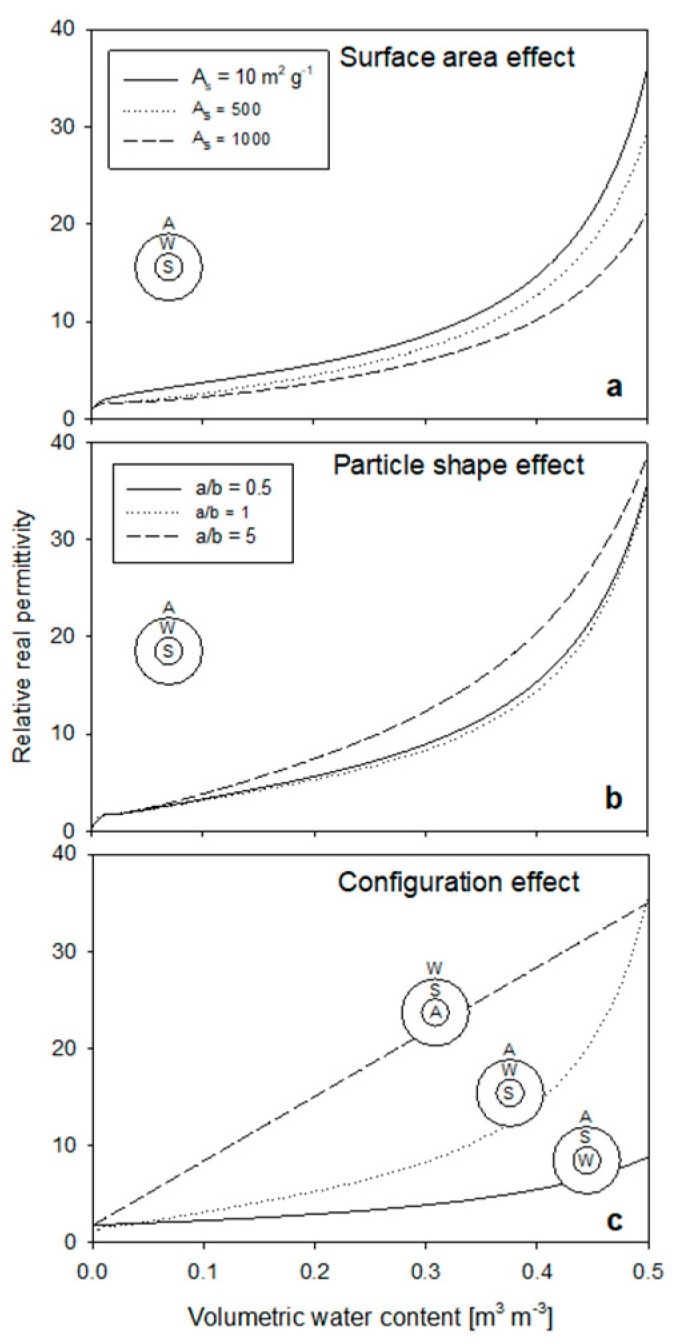
Three-phase confocal ellipsoid model (see [11] for details) demonstrating the influence of (**a**) surface area and (**b**) particle shape using a solid-water-air (SWA) configuration and demonstrating the influence of (**c**) phase configuration on the modeled permittivity.

Using Equation (8) with the SWA configuration the influence of surface area, particle shape and phase configuration are modeled in Figure 12. Surface area spans two orders of magnitude and the effects demonstrate the expected reduction in permittivity with increasing water-binding surface area (Figure 12a). The modeled surface area influence is also dependent upon the phase configuration, where a configuration of WSA would produce the minimum surface area influence based on the bound water model of Equation (23).

Particle shape effects are represented with aspect ratios ranging from 5 to 0.5 showing in this case a minimum permittivity for an aspect ratio of 1 (Figure 12b). Modeled shape-dependent permittivity is subject to the phase configuration. For example, a phase configuration of ASW produces the maximum permittivity for a spherical inclusion when compared to needle or disk-like objects [11]. The influence of phase configuration alone on the resulting permittivity is especially pronounced as shown in (Figure 12c), where the location of the water phase (i.e., core, shell or background) has a direct influence on the magnitude of the resultant permittivity due to the high dielectric constant of water (e.g., *ε*_w_ ≈ 80). From the six possible configurations of a three-phase system of concentric spheres, Friedman [11] demonstrated the significance of the position of the water phase on the resulting modeled permittivity, the field corn described in Section 3.1. Equation (8), with parameter coefficients listed in Table 8, produced minimum effective permittivities when water is in the core position and maximum when serving as background. In addition to the factors influencing permittivity demonstrated in Figure 12, measurement frequency, temperature, chemistry and bound-/free-water partitioning are among other factors of interest. Modeling the influence of these factors as well as isolating and identifying the extent or their respective effects should be the goal of future research.

#### 3.2.2. Modeling the Permittivity of High-Moisture Corn

**Figure 13:**
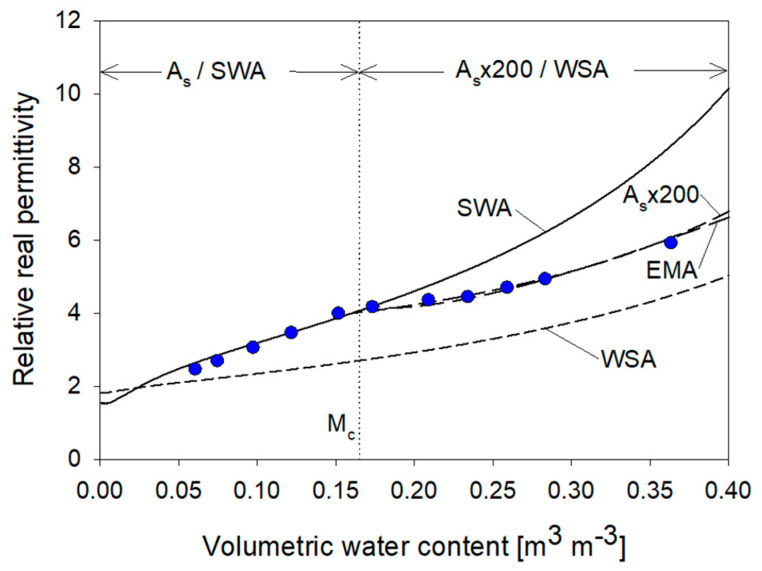
Measured permittivity of field corn at 200 MHz (digitized from [12]) modeled using surface area (*A*_s_ = 200 m^2^ g^−1^) and using a combination of phase configurations, SWA and WSA as described by Jones and Or [26]. The data were modeled by increasing specific surface area beyond the critical moisture content, Mc, and adding only the incremental water phase permittivity which required *A*_s_ = 40,000 m^2^ g^−1^ for fitting. Using a different approach, an effective medium approximation (EMA) was applied where all water below M_c_ was entirely configured as SWA and after *M*_c_ a phase configuration of WSA was used.

For modeling the permittivity of a moist cereal kernel, a configuration of solid-water-air (SWA) was selected. This configuration is applied to modeling the real component of the measured permittivity of the modeled result shown in Figure 13, which predicts the measured permittivity through the dry kernel region, after which the measured values fall between the SWA and WSA configurations. The measured data demonstrate a reduction in the rate of permittivity increase with increasing moisture near median water contents. The cause of the reduction in the rate of permittivity increase is not well understood, but several hypotheses can be made based on the water-phase within the kernel. One approach is to assume that additional surface area is exposed as grain and starch granules swell (shrink when drying; see Section 2.3.1), resulting in increased water binding capability. A decrease in the modeled SWA permittivity may result from bound water, which appears to be increasing with increased water content. To model this phenomenon, we computed *ε*_SWA_ up to the critical moisture content, *M*_c_ using *A*_s_ = 200 m^2^ g^−1^, after which the incremental dielectric constant of the water phase was added to *ε*_SWA_(*M*_c_) based on an immediate increase in *A*_s_ since we have no knowledge of how it may actually evolve. The reduced water content-permittivity relation is plotted for the SWA phase configuration in Figure 13 labeled *A*_s_ × 200.

For fitting the measured permittivity data, an unreasonably large value of *A*_s_ = 40,000 m^2^ g^−1^ was required. There are several possible reasons for such a large value of *A*_s_. One is that our simplified approach for modeling the water phase in the context given is not adequate and needs further refinement. Another suggestion comes out of the analysis of different phase configurations shown in Figure 12c. If water becomes more deeply embedded within the solid near M_c_, this would create a shift from a SWA dominated system to one where additional water is added in a WSA configuration. This second idea also has implications on the way surface area or bound water influences the permittivity since water in the core (WSA) has less of an impact on permittivity than water present in the shell or background phases.

The concept of multiple phase configurations has been used previously to represent dry and wet soils as a continuum (e.g., [11,165]) and may be used to combine the modeled SWA phase configuration with that of WSA also shown in Figure 13. This approach seems intuitive observing how the “wetter” data fall within the envelope formed by these two-phase configurations, suggesting water exists in both the core and shell phases described by the mixture model. Seeking a simplified approach, we partitioned all water up to *M*_c_ to the SWA phase configuration. Beyond M_c_, we assume that all additional water held within the grain is distributed to the WSA configuration. This partitioning is described as the fractional contribution of the WSA configuration, *f*_WSA_, and by that of the SWA phase configuration, *f*_SWA_, where *f*_WSA_ = 1 − *f*_SWA_ and *f*_SWA_ is given as:

(25) $$f_{\mathrm{swa}}=|\begin{array}{ccc} 1 & if & M_{v}<M_{c}\\ 1-\frac{M_{c}}{M_{v}} & if & M_{v}\geq M_{c} \end{array}\;$$

The resulting permittivity is derived from *ε*_SWA_ and *ε*_WSA_ which are combined using an effective medium approximation (EMA), treating both configurations symmetrically [165].

(26) $$\varepsilon_{\mathrm{ema}}={\left\{\frac{\left[f_{\mathrm{swa}}\left(\varepsilon_{\mathrm{wsa}}-2\varepsilon_{\mathrm{swa}}\right)+f_{\mathrm{wsa}}\left(\varepsilon_{\mathrm{swa}}-2\varepsilon_{\mathrm{wsa}}\right)\right]}{16}+\frac{\varepsilon_{\mathrm{swa}}\varepsilon_{\mathrm{wsa}}}{2}\right\}}^{0.5}-\left[\frac{f_{\mathrm{swa}}\left(\varepsilon_{\mathrm{wsa}}-2\varepsilon_{\mathrm{swa}}\right)+f_{\mathrm{wsa}}\left(\varepsilon_{\mathrm{swa}}-2\varepsilon_{\mathrm{wsa}}\right)}{4}\right]\;$$

From the resulting modeled permittivity of the corn, we observe that below M_c_, all water surrounds the solid phase as described by the SWA configuration. Above M_c_, the trend suggests that all of the additional water added becomes embedded within the solid matrix and indeed the permittivity data beyond *M*_c_ increase at a rate similar to the modeled WSA configuration. We suggest that both surface area evolution and phase configuration play roles in the phenomena presented in Figure 13. It appears from our analysis that phase configuration is the major contributor to the reduced rate of permittivity rise in the measured data. Further work is needed to clarify the nature of the water content-permittivity relationship of cereal grains and their constituents and particularly that of higher moisture grains such as corn.

#### 3.2.3. Ear Corn Moisture and Permittivity

**Figure 14:**
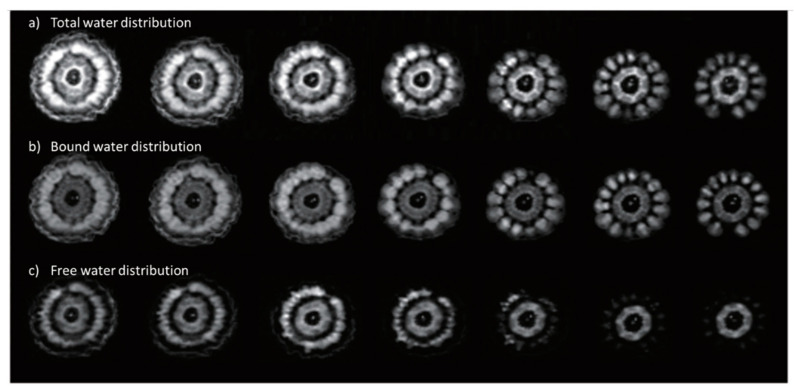
Daily grayscale cross-sectional magnetic resonance images of fresh ear corn (**a**) total-water, (**b**) bound-water and (**c**) free-water, (i.e., expressed as light color intensity) over a week-long drying process. Modified from Wang et al. [25].

**Figure 15:**
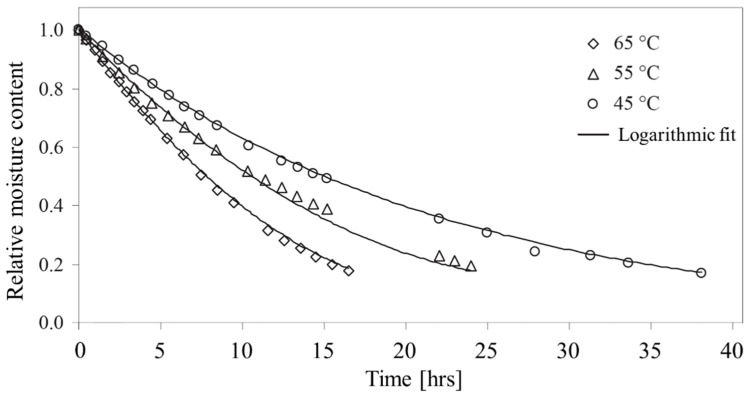
Ear-corn dry-basis relative moisture content as a function of drying time at 3 different temperatures. A logarithmic model was fitted to data yielding determination coefficients of 0.999 in all three cases. Modified from Corrêa et al. [170].

In addition to the drying corn kernels, the water content and subsequent drying of whole ear corn is of interest in the seed industry. The consideration of kernels on the corn ear introduces additional complication to the drying process as well as to the interpretation or modeling of the moisture content within the ear constituency. Figure 14 illustrates cross-sectional MRI images of an ear-corn sample taken daily, at the same location within the ear, over one week of drying [25]. Insightful here is the separation of total-, bound- and free-water distributions presented in Figure 14a–c, respectively. Much of the kernel free-water had evaporated after a week while water bound within the kernel had increased relative to the free-water from 60 percent to roughly 100 percent as illustrated [25]. In a different study, the dry-bases relative moisture content was defined as:

(27) $$\begin{array}{c} M_{\mathrm{dr}}=\left(\frac{M_{\mathrm{db}}-M_{e}}{M_{i}-M_{e}}\right) \end{array}$$

where the measured dry-basis moisture content, *M*_db_, was normalized using the initial moisture content, *M_i_*, and the final equilibrium moisture content, *M*_e_. Figure 15 presents corn ear drying over several days at 45, 55 and 65 °C. The resulting temporal drying data were described using a logarithmic function, which matched all three data sets with R^2^ = 0.999. The 20 °C higher temperature (65 °C) reduced the drying time by more than half compared to the 45 °C temperature, with a commensurate increase in the energy cost of drying.

**Figure 16:**
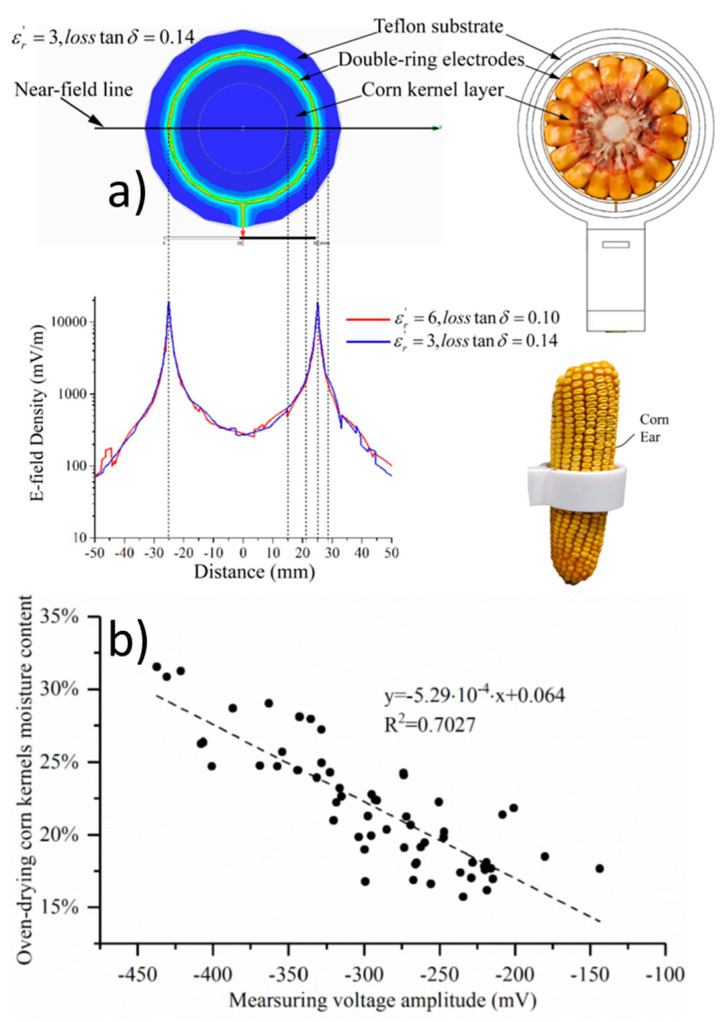
(**a**) Simulation results for the horizontal electric field distribution (along the near-field line) between the electrodes and the kernels/substrate for different kernel layer dielectric properties. Fan et al. designed a hand-held dual-ring sensor that surrounds the corn ear. (**b**) Ear kernel moisture content from oven-drying as a function of sensor output. Modified from [173].

Individual ears of corn drying in the field, prior to harvest, may be monitored periodically through sampling and oven drying. Even more convenient is the ability to take real-time measurements of ear moisture using hand-held moisture devices [171,172]. Figure 16 illustrates a double-ring sensing probe for non-destructive kernel moisture detection in intact corn ears [173]. The 160 MHz operating frequency of the active reflection bridge circuit provided a linear output of voltage with kernel permittivity and temperature. Figure 16a exhibits the magnitude of the electric field generated with peak sensitivity near the pair of rings at the kernel interface. Figure 16b presents the training data set with voltage output as a function of corn kernel oven dried moisture content with R^2^ of 0.703. Given the variable characteristics of corn ears and kernels, it is not surprising that the correlation coefficient of the calibrated instrument with moisture content ranging between 15.7–31.5 percent was only 0.83, yielding a moisture prediction error of 3.5 percent at the 95 percent confidence interval. While this type of handheld instrument provides a useful tool for monitoring in-field ear corn moisture, additional improvements are needed to arrive at the less than 1 percent moisture content resolution required for U.S. Federal measurement standards.

The concept of an instrumented “agent ear” illustrated in Figure 16 or even an “agent kernel” [174] whose moisture content is continuously monitored during the grain drying process is certainly appealing for both in-field and in-bin applications. Assuming the sensing component does not substantially impact the corn-ear or kernel drying rate relative to surrounding ears, the “agent” could provide both a representative measure of drying rate within the field or a drying bin as well as to provide estimates of threshold moisture for harvest, storage and marketing. Recent developments in the miniaturization of vector network analyzers and simulation software have created new opportunities for potentially portable, lightweight measurement devices yielding complex spectral permittivity in the near future [175].

## 4. Summary and Outlook

The primary factors affecting agricultural porous media permittivity measurements are the presence of both free and bound water, chemical composition, temperature, constituent shape and phase configuration, in addition to measurement frequency. Permittivity measurements in cereal grains and legumes are most significantly influenced by water content and status, where water bound to solid surfaces exhibits a reduced permittivity compared to free water. Both bound and free water exhibit temperature dependence, though their effects are inversely related. Distinguishing absolute water content using permittivity measurements is not straight forward because of the complex and varied composition and water binding character of different grains. The frequency dependence of permittivity measurements is a function of relaxation phenomena that are associated with constituent size (i.e., ion, molecule, atom) and with phase configuration relative to the applied electric field (i.e., parallel or series configuration). Constituent shape influences the permittivity according to dipole moments that likely occur throughout the hierarchy of the grain structure from the kernel to starch grains to the water and starch structures within.

The influence of the physical and chemical properties of cereals and legumes and of their constituents on the resulting permittivity are also of interest for improving the estimation of water binding and other elements of influence. Starch is a major constituent and comprises up to 70 percent of cereals followed by proteins at around 10 percent. Approximations for starch and protein surface area and assuming a monolayer coverage of water were presented and are useful for predicting bound water content in these materials. Grain packing is a function of grain shape and moisture content. Using a physically based water content-dependent relation for kernel density, expressions for porosity and bulk density were derived. In general, water within grains and legumes at below harvest moisture levels (i.e., 0.24 wb) appears to be entirely bound and exhibits temperature-dependent permittivity behavior where permittivity increases with increasing temperature (opposite to that of free water). Because activation energies for water bound in cereals and their constituents are similar to values found in fine-textured and high surface area soils and other porous media, models developed for water binding on clay and mineral surfaces may be applicable to carbohydrate and protein water binding as well.

Dielectric mixture models have been derived based a variety of assumptions regarding particle background interactions for 2, 3 and 4-phase systems. Factors affecting permittivity such as grain shape can be modeled using simplified geometries and assumptions regarding the particle-field orientation. The temperature-dependent permittivity of the water phase was modeled and may be incorporated into mixture theories as a single phase. A two-phase mixture model may provide a general approximation of the permittivity for cereal grains or legumes as a function of moisture content. The added capacity of a three-phase mixture model allows analysis of factors influencing permittivity such as water-binding surface area, grain shape and phase configuration. Anomalous water content-permittivity behavior noted in corn starch and whole kernel corn appears to be related to water binding or phase configuration within the grain. Further work is required to separate these and other effects and to understand their relative influence on permittivity measurements. Dielectric mixture models provide a physically-based and more generalized approach for modeling some of the factors influencing the permittivity of cereal grains used for moisture content prediction.

Despite the recent availability of grain moisture meters at local retailers, low-cost sensors are often associated with low quality measurements. Accurate determination of moisture content in porous materials will remain a significant challenge going forward given the variety of porous medium physical and chemical properties coupled with environmental factors that also influence measurements, all important inputs for accuracy. The monumental development of a unified grain moisture measurement algorithm based on such inputs, brought substantial improvement to cereal grain moisture determination at 149 MHz [20,21]. However, further improvements using smart sensing technologies are likely as electrical components and circuitry and analysis and computational algorithms are further improved. A good example of how fast technology has advanced are the development of many low cost—miniaturized network analyzers that literally fit in your hand and which will soon provide high quality measurements as highly portable devices [175]. The array of non-destructive methods available today provide a wide spectrum of information (e.g., chemometrics) that may be coupled with artificial intelligence and machine learning. Those methods include, near-infrared (NIR), infrared (IR), Raman spectroscopy and fluorescence spectroscopy, along with colorimetric sensor array (CSA), imaging-based techniques. The data fusion and machine learning strategies create promising techniques for the quality, authenticity and discrimination of agricultural grains [176]. Tapping into hyperspectral imaging has shown potential to rapidly classify cereal grains based on their variety, kernel hardness, geographical origin, etc., with additional insights to come based on its potential for rapid single-kernel analysis [177]. Novel processing and treatment techniques such as high hydrostatic pressure improve grain quality and longevity in storage [178,179]. Low-field nuclear magnetic resonance (NMR) and magnetic resonance imaging (MRI) have been applied to monitor the migration and distribution of moisture in ear corn [25].

## Figures and Tables

**Table 1:** Constituent properties of corn, rice and wheat obtained at 12–16% moisture content on a wet-basis (wb) and typical harvest and storage moisture contents.

Property	Corn	Rice	Wheat	Soybean ^†^	Lentil ^†^
Starch (%)	63.6	64.3	69.7	31.6	61.2
Protein (%)	9.8	7.3	10.6	34.3	24.7
Fat (%)	4.9	2.2	1.9	18.7	1.0
Fiber (%)	2.0	0.8	1.0	3.8	4.1
Maximum harvest moisture ^‡^	25	38	20		
Optimum harvest moisture ^‡^	23	22	18		
Storage moisture > 1 year ^‡^	13	13	13	11	14

Data from [99] pp. 23–25 and sources therein. ^†^ Source—Augustin and Klein [100]. ^‡^ % wet-basis (wb) moisture.

**Table 2:** Corn, rice, wheat and potato starch properties.

	Corn	Rice	Wheat	Potato
Starch granule density (g cm^−3^) ^†^	1.517	1.510	1.542	1.511
Ext. surface area, *A*_e_ (m^2^ g) ^‡^	0.031	0.80	0.19	0.085
Diameter referencing *A*_e_ (μm)	12	4.6	19	43
Starch bulk density (g cm^−3^) ^§^	0.810	0.678	0.790	
Starch dielectric constant ^§^	2.74	1.25	2.42	

^†^ [107]; ^‡^ [102]; ^§^ dry granular starch measured at 2.45 GHz [75].

**Table 3:** Protein content in different cereal grains [114] and calculated specific surface areas (*A*_s_) of the protein alone and with respect to the whole kernel.

	Protein and Associated Molecular Weight				*A*_s_ Protein Only	Kernel Protein Content	*A*_s_ within Kernel
	Albumin	Globulin	Prolamin	Glutelin			
	12,000 ^†^	320,000 ^†^	21,000 ^†^	1 × 10^6 ‡^			
**Cereal**	**% Protein in Each Seed Variety**				**m^2^ g** **^−1^**	**%**	**m^2^ g** **^−1 §^**
Barley normal	13	12	52	23	1910	10.9 ^¶^	209
Maize normal	4	2	55	39	1730	10	173
Millet, Pearl	13.2	9.4	40	28	1810	16	289
Oat	1	78	16	5	1210	11	134
Rice	5	10	5	80	900	9	81
Rye	10	10	40	40	1630	11.5	187
Sorghum Normal	8	8	52	32	1790	11	197
Triticale	26.4	6.5	24.4	36.3	1790	15	268
Wheat	5	10	69	16	2030	12.2 ^¶^	247

^†^ [115]; ^‡^ [116]; ^§^ average of min and max values; ^¶^ [117].

**Table 4:** Physical properties of corn, rice and wheat at 12–16% moisture content (wb).

Property	Corn	Rice	Wheat	Soybean ^§^
Length (mm) ^†^	8–17	5–10	5–8	5.4
Width (mm) ^†^	5–15	1.5–5	2.5–4.5	6.6
Aspect ratio, *a*/*b*	1.6–1.1	3.3–2.0	1.8–2.0	0.81
Kernel weight (mg) ^†^	150–600	23–27	30–45	120
Bulk density, *ρ*_b_ (g cm^−3^) ^†^	0.745	0.590	0.805	0.721
Bulk porosity, *φ*_b_ ^†^	0.40	0.48	0.41	0.382
Kernel density, *ρ*_k_ (g cm^3^) ^‡^	1.24	1.13	1.36	1.17
Avg. solid density *ρ*_s_ (g cm^−3^) ^¶^	1.45	1.43	1.47	1.34
Kernel specific surface area (m^2^ m^−3^) ^†^	784	1132	1181	870

^†^ Data from Brooker et al. [99] pp. 23–25 and sources therein. ^‡^ Computed from bulk density and porosity data. ^§^ Data at 14% (wb) moisture content from Deshpande et al. [124]. ^¶^ Computed from Table 1 constituent fractions and densities taken from Peleg [125].

**Table 5:** Crossover frequencies of different materials observed from water content and temperature variations as a function of measurement frequency.

Material	Crossover Frequency	Temperature Range	Reference
	MHz	°C	
5 soils	10–110	5–45	Chen and Or, [139]
Chicken Breast Muscle	100–200	5–85	Zhuang, [141]
Whey protein gel	100	5–95	Nelson, [142]
Navel orange	50	5–65	Nelson, [140]
Russett Burbank potato	70	5–65	Nelson, [140]
Red delicious apple	23	5–65	Nelson, [140]
Avocado	90	5–65	Nelson, [140]
Banana	50	5–65	Nelson, [140]
Cantaloupe	80	5–65	Nelson, [140]
Carrot	120	5–65	Nelson, [140]
Cucumber	16	5–65	Nelson, [140]
Thompson seedless grape	23	5–65	Nelson, [140]

**Table 6:** Estimated specific surface area and water content (db) of mono- and multi-layered bound water measurements in various proteins, starches and flours.

Molecular Layers		1	1	2–3
Material	Method	*M*_db_	*A*_s_	*M*_db_
		(g g^−1^)	(m^2^ g^−1^)	(g g^−1^)
BSA [62]		0.25	633	
34 protein avg. [109]		0.28	709	
Lysozyme [145]	NMR			0.33
Myoglobin [145]	NMR			0.43
Ovalbumin [145]	NMR			0.33
Hemoglobin [145]	NMR			0.43
β-casein [62]	Admittance	0.24	607	
Myoglobin [62]	Admittance	0.22	557	
Hemoglobin [74]	DRS			0.20
Wheat starch [143]	NMR	0.17	430	
Potato starch [146]				0.30
Corn starch [113]	TSDC	0.15	380	
Corn starch [147]	NMR			0.35 ^†^
Corn starch [148]	Dilatometry			0.35
Corn starch [149]	NMR			0.32
Corn starch [144]	NMR	0.19	481	
Maize [150]	Thermodyn	0.08	202	0.25
Cracker dough [151]	DSC			0.25 ^†^
Bean flour [152]	TSDC	0.17	430	
Soy flour [113]	NMR	0.39	987	
Wheat flour [152]	TSDC	0.11	278	
Wheat flour [147]	NMR			0.49 ^†^

^†^ Converted from wet to dry-basis (*M*_db_ = *M*_wb_/(1 − *M*_wb_)), NMR = nuclear magnetic resonance; DRS = dielectric relaxation spectroscopy; TSDC = thermally stimulated depolarization currents; DSC = differential scanning calorimetry.

**Table 7:** Calculated specific surface area (*A*_s_) and cumulative specific surface area based on corn starch constituent geometries approximated as spheres or cylinders.

	Assumed	Axial	Cylinder		Cumulative
Structure Level	Geometry	Radius ^†^	Radius ^†^	*A*_s_	*A*_s_
		m	m	m^2^ g^−^^1^	m^2^ g^−^^1^
Starch grain	sphere	1.0 × 10^−6^		2	2
Large blocklets	sphere	2.5 × 10^−8^		80	82
Small blocklets	sphere	1.0 × 10^−8^		200	280
Amylopectin Cluster	cylinder	5.0 × 10^−9^	5.0 × 10^−9^	260	540
Amylopectin/Amylose unit cell	cylinder	3.0 × 10^−9^	8.0 × 10^−10^	1650	2190

^†^ Gallant et al. [87]; Constituent density = 1.52 from Table 2.

**Table 8:** Equation variables used for modeling permittivities plotted in Figure 11, Figure 12 and Figure 13.

		Corn, Wheat, Soy Beans		Corn	Corn	
Variable	Used in Equation Number	Figure 11	Figure 12	Figure 13 (*A*_s_)	Figure 13 (EMA)	Units
*a*/*b*	Equations (5) and (19)	1.4, 2.5, 0.81	1 ^†^	1.4	1.4	m m^−^^1^
*ρ*_s_, (Table 4)	Equations (17) and (20)	1450 1470 1340	1500	1450	1450	kg m^−^^3^
*ρ*_b_	Equation (14)	*f*(*θ*) Equation (20)	750 ^†^	612	612	kg m^−^^3^
*ε*_a_	Equations (7) and (9)	1	1	1	1	
*ε*_s_	Equations (7) and (9)	3.3, 3.8, 4	2.5	2.5	2.5	
*ε*_w_	Equations (7) and (9)					
*T*	Equations (2) and (4)	25	25	25	25	°C
f*	Equation (4)	1	1	1	1	GHz
*r*	Equation (4)	2.5	2.5	2.5	2.5	Å
*M*_c_	Equation (24)			0.18	0.16	
*A*_s_	Equations (3) and (23)	200, 280, 990	200 ^†^	200	200	m^2^ g^−^^1^

^†^ or as specified in Figure 12.

**Table 9:** Calculation of volumetric fractions of core (*ε*_2_), shell (*ε*_1_) and background (*ε*_0_).

Description	Designation	Phase Configuration		
		WSA	SWA	ASW
Core volume (cm^3^)	*V*2	4π*abc*/3	*M*_v_ *V*_1_	*V*_1_(*ϕ*_b_−*M*_v_)
Shell volume (cm^3^)	*V*1	*M*_v_*V*_0_ + *V*_2_	4π*abc*/3	4π*abc*/3
Background volume (cm^3^)	*V*_0_	*V*_2_/(1−*ϕ*_b_)	*V*_1_(1 + *ϕ*_b_−*M*_v_)	*V*_1_(1 + *M*_v_)
Core vol. fraction	*ϕ*2	*V*_2_/*V*_0_	*V*_2_/*V*_0_	*V*_2_/*V*_0_
Shell vol. fraction	*ϕ*1	(*V*_1_ − *V*_2_)/*V*_0_	(*V*_1_ − *V*_2_)/*V*_0_	(*V*_1_ − *V*_2_)/*V*_0_

## Data Availability

All data presented were digitized or reproduced from sources cited.
